# Conformational Dynamics, Ligand Binding and Effects of Mutations in NirE an S-Adenosyl-L-Methionine Dependent Methyltransferase

**DOI:** 10.1038/srep20107

**Published:** 2016-01-29

**Authors:** Warispreet Singh, Tatyana G. Karabencheva-Christova, Gary W. Black, Jon Ainsley, Lynn Dover, Christo Z. Christov

**Affiliations:** 1 Department of Applied Sciences, Faculty of Health and Life Sciences, Northumbria University, Newcastle upon Tyne, NE1 8ST, United Kingdom

## Abstract

Heme d1, a vital tetrapyrrol involved in the denitrification processes is synthesized from its precursor molecule precorrin-2 in a chemical reaction catalysed by an S-adenosyl-L-methionine (SAM) dependent Methyltransferase (NirE). The NirE enzyme catalyses the transfer of a methyl group from the SAM to uroporphyrinogen III and serves as a novel potential drug target for the pharmaceutical industry. An important insight into the structure-activity relationships of NirE has been revealed by elucidating its crystal structure, but there is still no understanding about how conformational flexibility influences structure, cofactor and substrate binding by the enzyme as well as the structural effects of mutations of residues involved in binding and catalysis. In order to provide this missing but very important information we performed a comprehensive atomistic molecular dynamics study which revealed that i) the binding of the substrate contributes to the stabilization of the structure of the full complex; ii) conformational changes influence the orientation of the pyrrole rings in the substrate, iii) more open conformation of enzyme active site to accommodate the substrate as an outcome of conformational motions; and iv) the mutations of binding and active site residues lead to sensitive structural changes which influence binding and catalysis.

Uroporphyrinogen III (uro’gen III or UP2) acts as a common scaffold for the synthesis of diverse tetrapyrroles such as chlorophylls^1^, cobalamins, siroheme, phytochromobilin, heme *d*1, and coenzyme F430^2^,^3^. Heme d1 is an iron-containing dioxo-isobacteriochlorin which acts as a cofactor for cytochrome *cd*1 nitrite reductase enzyme^4^. Cytochrome *cd*1 nitrite reductase is the only enzyme in which heme d1 is a cofactor and where it functions as a site for nitrite reduction^5^ i.e. reduction of nitrite to nitric oxide and water^6^,^7^. This denitrification process is a respiratory mechanism for many bacteria including human pathogen *Pseudomonas aeruginosa*, and thus represents a potential drug target^8^,^9^. Direct inhibitors targeting specific enzymatic processes of *P. aeroginonsa* have distinct advantages over general antibiotics; they reduce the selection pressure towards antibiotic resistance in bacterial populations and can be used in combination with antibiotics to increase effectiveness and lower dosage requirements. With the rise of more and more antibiotic resistant bacteria it has become imperative to design new therapies that reduce the likelihood of creating multiple antibiotic resistant bacterial strains^10^. The synthesis of heme d1 proceeds via precorrin-2 which is the product of two methyl group transfers to uro’gen III^11^. The methyl groups are transferred from *S*-adenosyl-L-methionine (SAM) to uro’gen III by SAM-dependent Uro’gen III MethylTransferase (SUMT) called NirE^4^,^11^. The crystal structure of *P. aeruginosa* NirE in complex with its substrate uro’gen III and the reaction by-product SAH (*S*-Adenosyl-L-homocysteine) was solved recently^12^ (Figs 1 and 2). The NirE enzyme is a homodimer; each monomer consists of two domains A and B which are connected by a shorter linker region of four residues length. The active site pocket of NirE is located between the two domains of each monomer however residues from both monomers contribute to each active site^12^. NirE is subject to substrate inhibition at high concentrations of uro’gen III and product inhibition at high concentrations of SAH^13^,^14^. The crystal structure describes the detailed binding of UP2 indicating that it is exposed to solvent and bound loosely in the active site. However SAH binds tightly and is located in the deep interior pocket of the active site^12^. Proteins are large flexible molecules and conformational dynamics is a fundamental property which correlates proteins structure and functions^15^. Although the crystal structure of NirE reveals the important atomistic details of the enzyme, its ligand binding and possible catalytic mechanism, it does not relate how conformational flexibility and dynamics and the effects of mutations might influence key interactions with substrate and cofactor. In order to understand how structural plasticity might influence the structure-function relationships of NirE, we performed 50 ns atomistic (AT) molecular dynamics (MD) simulations on the wild-type full complex NirE, containing the apoenzyme, the cofactor and the substrate (WT FC), its mutant forms, the apoenzyme (APO), built from monomer A and B, the complex between the enzyme and the cofactor (EC), and the complex with the substrate (ES).

## Results

### Overall Stability and Flexibility of NirE Structures

Overall stability of the WTFC structure was assessed by considering the Root Mean Square Deviation (RMSDs) of Cα atoms during the MD simulations (Fig. 3A). The system equilibrated after 12ns, multiple runs of the WT FC trajectory showed an average RMSD value of 2.8 Å (Table S2, Figure S1). We considered the relative flexibility of secondary structural elements by class (α helices, β sheets and loops); beta sheets maintained the lowest RMSD value of 1.5 Å along the 50 ns trajectory compared to alpha helices (2.14 Å) and loops (2.9 Å) (Figure S2). The high RMSD values of the loop regions are a common feature of many proteins such as TPST-2 (Tyrosylprotein Sulfotransferase) where loops displayed the highest RMSD values compared to alpha helices and beta sheets. The RMSD profile of individual domains of monomer A indicates that the domain B is more stable compared to domain A; even though both domains have the same secondary structural topology (Figure S3). The reason for the higher stability of domain B in respect to domain A is that majority of the residues which participates in stabilizing interactions with the substrate and cofactor such as R*149, H161, M186 etc. are located in domain B. The RMSD profile of the linker region (domain A and domain B, here of monomer A) showed a reltively stable RMSD; however there was overall increase in the RMSD after 30 ns which could be attributed to the change of Cα angle from 135° to 140° between the residues (129, 132 and 134) of the linker region (Figure S4). The RMSD profile of the mutants equilibrates around 12 ns apart from E114Q and H161F mutants which equilibrated around 18 ns and 10 ns respectively (Fig. 3A). The average RMSD value of all Cα atoms of WT FC is ~ 3.2 Å, whereas in mutants its ranges from 2.4 Å in E114Q and R*149 deprotonated to 3.1 Å in the R111K mutant (Table S3, Fig. 3A and Figure S5).

The Root Mean Square Fluctuations (RMSF) of WT FC (Fig. 3B) showed that the loop between β3 and the D alpha helix (residues 70–80) has high levels of fluctuation. This loop was missing from the reported crystal structure^12^ and was modelled for MD simulations, indicating it to be a particularly flexible component of the protein despite its proximity to substrate UP2 in the crystal structure.. The short loop region between E alpha helix and β5 (residue 121–125) also displayed increased flexibility compared to other residues in the RMSF plot. The loop region and G alpha helix encompassing residues 163–179 between β6 and β7 also showed high flexibility. The residues forming this loop make important interactions especially with the side chain of the UP2 substrate during the MD simulation. The residues 214–220 that form the loop between β8 and β9 make interactions with the adenine ring of SAM also revealed greater flexibility than the majority of other residue in WT FC. The loop between β9 and I alpha helix also showed increased fluctuations in WT FC. The basal level of fluctuation of the WTFC was considered to be 1.1 Å, 53% of RMSFs in WTFC are <1.1 Å and 47% of the residue’s RMSFs are >1.1 Å. The mutants RMSF >1.1 Å range from 29% (E114Q) to 45% (R*149 de-protonated) (Figure S6). The detailed analysis of the residues of the mutants which showed increased or decreased RMSF’s relative to WT FC are represented in Table S4. The substituted residues in the mutants used in this study are all located in close proximity of the substrate UP2 and are mainly involved in substrate binding apart from M186 which is involved in both substrate and cofactor binding. It was evident from the RMSFs of all the mutants in comparison to WT FC that the substrate binding region was clearly affected. In particular there were increased fluctuations of substrate binding residues (16–170), located on the loop between β6 and G helix in every mutant (Figure S7 and Table S4). This loop showed high flexibility in WT FC as well and was found to be missing from the crystal structure^12^ due to its high flexibility. The mutants R51K, R*149K, M186K, G189K and R111K show increased fluctuations of the residues located on the C alpha helix and loop between C alpha helix and β3. This region is also involved in substrate binding in WT FC. The residues (70–80) of the loop located between β3 and D alpha helix showed increased flexibility in all the mutants in comparison to WT FC and these residues were also involved in the substrate binding and were also missing in the crystal structure^12^ (Figure S8). The mutations increased the local fluctuations of the substrate binding residues in NirE. Many substrate binding residues (R51, R111, H161, M189, R*149 and G189) in the WT FC aremainly located on the loop region of the enzyme and the methyl donor binding (SAM) residues with the exception of (M186, D105, I108 and 217) were mainly located on the alpha helices and beta sheets of the protein, further confirmingthe relatively high flexibility of the substrate binding residues.

### Cofactor Interactions between Substrate and Protein

**Figure 2:**
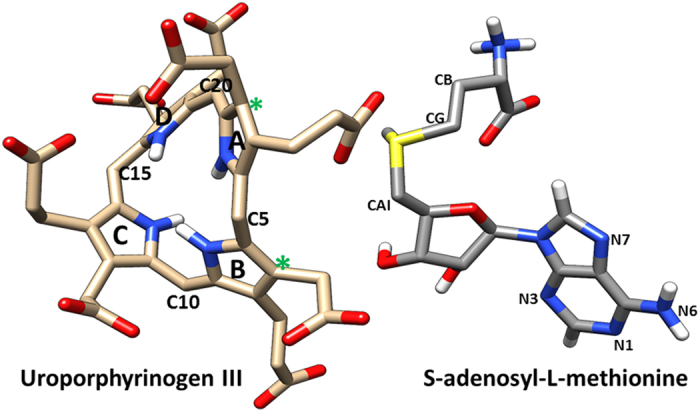
The substrate Uro’gen III pyrrole ring system from (A–D), the C20 carbon atom is a potential proton abstraction site on UP2 and the asterisks represents potential methyl group acceptor sites in the substrate from SAH. The images were drawn using UCSF Chimera^33^ in sticks representation.

**Figure 3:**
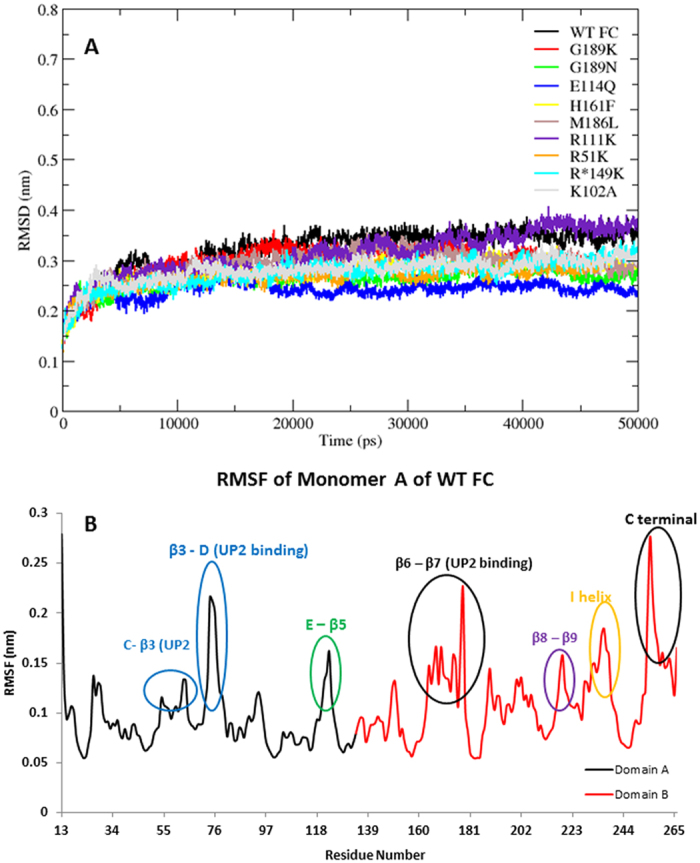
(**A**) The Root Mean Square deviation (RMSD) of all Cα atoms of wild type NirE (WTFC) and the mutants for 50 ns trajectory, (**B**) RMSF of all Cα atoms of residues of WT FC for 50ns trajectory. The RMSD plot was drawn using the Xmgrace plotting tool.

The time evaluation of interactions between the methyl donor (SAM) and substrate (UP2) were analysed for WT FC. The propionate side chain of ring A of UP2 formed electrostatic interactions with the positively charged sulphur atom of SAM for the entire length of simulation (average distance of 3.1 Å, Figure S9). The distance between the methyl group of the SAM and the potential methyl acceptor sites on ring A and B of the substrate UP2 (Fig. 2) showed average distances of 6.0 and 6.3 Å respectively. The angle between the sulphur, methyl group and methyl acceptor sites on the substrate was sampled over the 50 ns trajectory and found to be on average 93.7° (Figure S10). The addition of the transferred methyl group to the cofactor (SAM) resulted in the appearance of a new hydrophobic cluster which was not present in the crystal structure of NirE^12^. This newly formed hydrophobic cluster consisting of M186, Y185 and F109 residues stabilized the methyl group of the SAM with an average distance of 4.3, 4.2 and 4.0 Å respectively (Figure S11, Table S7). These interactions likely play a significant role in maintaining the correct orientation of the methyl group in the active site of NirE for the methyl transferase reaction.

We considered that our inclusion of SAM in these simulations could reveal a different pattern of interaction of the methyl donor than those evident with the spent donor SAH in the crystal structure. The hydrogen bonds between the backbones of I108 and D105 with the amino group (NH^3+^) of the SAM existing in the crystal structure^12^ are stable during the MD simulation of WT FC. However the hydrogen bonds between the side chain of D105 and the amino group (NH^3+^) of SAM were not maintained after 30 ns in the simulation due to an increase in distance (Figure S12). The crystal structure^12^ describes the presence of hydrogen bonds between the carboxylate of SAM with sidechain of T133 and between the backbone of A134 and D105. In this MD study, the carboxylate group of the SAM is hydrogen bonded to the side chain of T133 as described in the crystal structure^12^; however a new interaction between the side chain of Y185 and carboxylate of SAM, not present in the crystal structure appeared. The backbone of A134 made weak interactions with the carboxylate of SAM and with the Y185 sidechain. This interaction positions the side chain of Y185 to establish its own interaction with the carboxylate group of the SAM. The backbone of D105 does not interact with the carboxylate group of SAM, in contrast to the SAH-bound crystal structure (Figure S13). The side chain of Y185 interacts with the dihydroxyoxolan ring of SAM with an average distance of 3.2 Å. In our simulation the carboxylate side chain of SAM forms an intramolecular interaction with the positively charged sulfur atom (Figure S14). The crystal structure^12^ suggests stabilization of the adenine ring by hydrogen bonds with G215 and P27. In our MD simulations neither residue interacts with the adenine ring, instead P27 makes hydrophobic interactions to A134 and L32 residues. In our simulation a completely new set of interactions of residues C138, P242, V212, Q214, Q217 and M186 stabilized the adenine ring by hydrogen bonds (Figure S15 and S16). L244 (3.3 Å, average distance) also interacts with the adenine ring of SAM (Table S5) (Figure S17). The side chain of P242, A134 and Y185 also made hydrophobic interactions with the adenine ring of the cofactor as in the crystal structure (Figure S18).

### Substrate interactions with the Protein

The crystal structure^12^ shows that the substrate UP2 adopts a twisted two-up and two-down conformation of the pyrrole rings, however in the MD simulation three out of four pyrrole rings move down and one ring takes an upward orientation. Initially, ring A and C were pointing downwards and ring B and D pointing upwards as described in the crystal structure. However during the course of the simulation ring B which was in an upward conformation shifts to point downward. The ring D remains in the upward conformation as described in the crystal structure. The dihedral angle for the four rings is plotted in Figure S18 and changes little over time with an average fluctuation of −10°. There are two water molecules localized in the vicinity of the sulfur of the cofactor and the propionate side chain of ring A indicated by two distinct peaks around distances of 3   and 4 Å (Figure S19) which might be related to catalysis. The crystal structure^12^ shows that the side chain of R*149 makes polar contacts with the guanidino group of R111, however in MD studies the R111 makes electrostatic interactions with the side chain of the acetate group of ring A. The side chain of R*149s drift away on average 8.9 Å from the side chain of the R111 during the MD simulation due to positive charge repulsion of the guanidino group (Figure S20). The R51 side chain also makes a very brief contact with the sidechain of the acetate group of ring A of UP2. Interestingly the aliphatic side chain of R51 makes hydrophobic interactions with the aliphatic portion of propionate side chain (ring A) and therefore stabilizes it to make stable interactions with the sulfur of the cofactor SAM (Figure S21). The crystal structure^12^ indicates the hydrogen bond between the backbone of G189 and propionate side chain of ring B which was not maintained in the trajectory (Figure S22). The side chain of R51 also does not interact with the acetate side chain of ring B of UP2 (Figure S23). The imidazole ring of H161 in the crystal structure^12^ interacts with the pyrrole ring C of UP2 by hydrophobic interactions which was not found to be stable in the MD simulation. The propionate side chain of ring C made only one hydrophobic interaction with the Q163 side chain with an average distance of 3.9 Å. The side chain of Q163 stabilized the propionate side chain of ring D by hydrogen bond with an average occupancy of 44% during the simulation (Table S5) which was not mentioned in the crystal structure (Figure S24, Figure S25) and suggests a possible role of Q163 in substrate binding and stabilization. The new set of hydrophobic interactions involving the T159 and L162 side chains emerged during the simulation to stabilize the propionate side chain of ring D with an average distance of 4.0 and 4.4 Å respectively (Figure S25, Table S6). The crystal structure^12^ reveals that the side chains of R111 and R*149 make hydrogen bonds with the acetate side chain of the ring D of substrate, while in MD the R*149 they did not make any stable interactions with any of the side chains of ring D. In the simulation the backbone of R111 along with G110 made hydrogen bonds with the acetate side chain of ring D. However due to the high flexibility of the acetate side chain the interactions of R111 and G110 to the acetate side chain of ring D, the hydrogen bonds broke after 15 to 20ns (Figure S26). In the crystal structure^12^ the sulfur of M186 makes a hydrogen bond with the NH group of the pyrrole rings A, and C, which was not maintained in the MD trajectory (Figure S27); however the side chain of M186 did participate in the hydrophobic interactions with the acetate side chain of ring D.

#### Conformational Effects of R111 and R*149 Deprotonations

**Figure 4:**
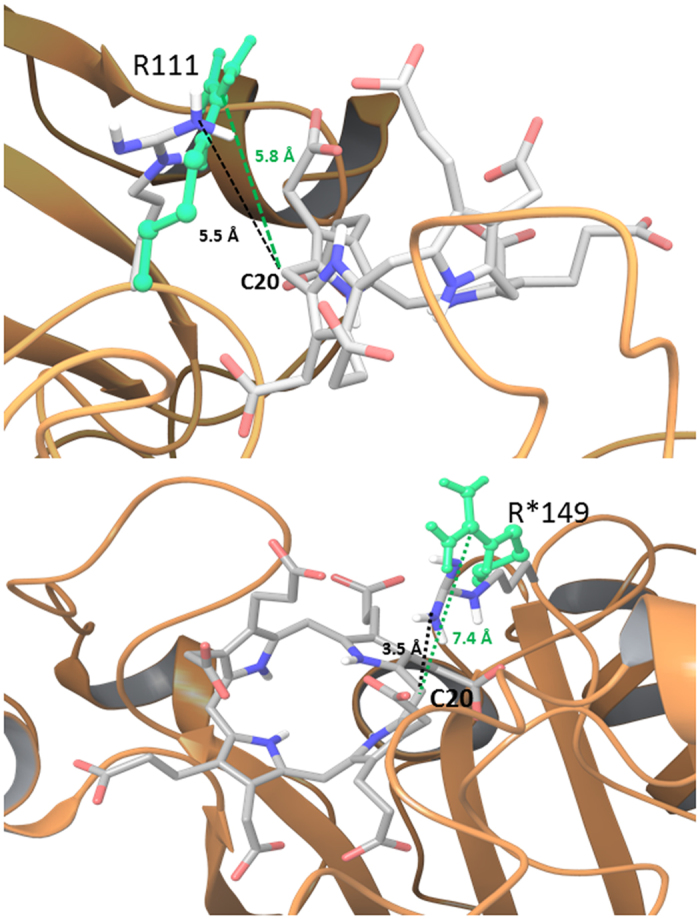
(A) The deprotonated R111 in comparison to protonated R111 to C20 of UP2, (B) The deprotonated R*149 in comparison to protonated R*149 to C20 of UP2. The distances shown in the figure are averaged over the entire length of 50 ns trajectory.

In the crystal structure^12^ the arginine residues R111 and R*149 in their deprotonated states, were proposed to be involved in proton abstraction from the C20 (potential proton abstraction site) of the substrate that initiates the methyl transfer reaction and E114 and E115, were proposed to stabilize arginine deprotonated state. The authors asserted that while arginine is not a good base, there are some enzymes where it might act as a base^16^ and further suggest that upon interaction with nearby Glu115, Glu114 and substrate carboxylates, one of the two arginines could undergo deprotonation and to act as a proton abstractor and supplemented their hypothesis by the kinetic measurements of the mutants of R111 and R149*^12^. Understanding that the idea of deprotonated arginines lacks strong supporting evidence we still wished to provide some modelling insight into this hypothesis, namely to simulate how the structure of the ES complex would look like if one by one R111 and R*149 were deprotonated and how this would affect the flexibility of the active site. In order to explore the effect of the deprotonation of R111 and R*149, and their interactions we performed MD simulations with each of the two arginines in their deprotonated states and compared the results to their protonated forms. In the MD simulation of the WT FC with both R111 and R*149 protonated, these residues are on average 5.8 Å and 7.4 Å away from the C20 site compared to 5.5 Å and 3.8 Å respectively in the minimized crystal structure (Figure S28). R111 makes stable interaction with the C20 of UP2 whilst the interactions of R*149 are highly flexible in WT FC. R111 is also involved in stabilization of acetate side chain of ring A and D via salt bridges and hydrogen bond respectively, however E114 made no significant interactions with either R111 or R*149 residues in WTFC (Figure S29). In the MD simulation where R111 was deprotonated (R*149 kept protonated), its distance to C20 atom of UP2 was not dramatically affected. The average distance from deprotonated R111 side chain to C20 is 5.5 Å, compared to 5.8 Å in WT FC (Fig. 4A), however there was a change in the distance of R*149 (in its protonated state) to C20 to 3.6 Å and this interaction was stable throughout the 50ns trajectory. The side chain of R*149 (in its protonated state) also made hydrogen bonds with the acetate side chain of ring D and with E114 which were not seen in the WT FC (Figure S30).

In the MD simulation where R*149 was deprotonated (with R111 in its protonated state) R*149 makes a stable interaction with an average distance of 3.5 Å to C20 of UP2 (Fig. 4B). However the distance between the R111 side chain and the C20 has increased in this simulation to an average of 7 Å as compared to 5.5 Å in R111 deprotonated setup. This increase in distance was due to the fact that the R111 residue made stable interactions with the E114 residue (Figure S31). The mutation of R*149 completely abrogated the enzyme activity indicating an indispensable role in catalysis and the mutation of R111 reduces the enzyme activity of wild type NirE by ~94%. Whilst we found some slightly closer distances for R*149 (Figure S32, Table S7) our results themselves cannot provide strong enough support in favor of the hypothesis for deprotonated arginines and the proposal would need further experimental and computational validation.

### Effects of Mutations of Residues involved in Binding and Catalysis

The mutation R51K reduced the enzyme activity by 60%, and slightly decreases SAM binding, which led us to conclude that R51 was mainly involved in substrate UP2 coordination^12^. In our MD studies, R51 was mainly involved in stabilization of the propionate side chain of ring A by hydrophobic interactions with an average distance of 4.1 Å. In the mutant R51K, the side chain of K51 makes stable electrostatic interactions with the acetate side chain of ring A, however there were no hydrophobic interactions and the average distance moved out to 5.7 Å between the side chain of K51 and the propionate side chain of ring A (Figure S33). Interestingly the conformations of the rings of UP2 are all in upward orientation in contrast to WT FC where three rings were downwards and one ring upwards. The RMSF of the loop region between the β2-C and β4-E showed increased fluctuations in the R51K mutant in respect to WT FC. The residues in vicinity of K51 (defined as ±10 residues away) in the R51K mutant showed increased fluctuations in comparison to WT FC. Interestingly the residues which are involved in cofactor binding and substrate binding present on the loop between β4-E (103–115) also showed increased fluctuations, indicating the long range effect of mutation on distant secondary structures in NirE (Figure S34).

R111K reduces the enzyme activity by 94% and also slightly affects the SAM binding with respect to wild type NirE^12^. In the MD studies, the side chain of R111 stabilizes the UP2 ring A acetate side chain by electrostatic interactions. The mutant R111K however made very unstable interactions with an average distance of 5 Å between the acetate of ring A of UP2 (Figure S35). The RMSFs of the residues in the vicinity (defined as **±**10 residues away) of K111 in the mutant R111K showed a significant increase in fluctuations. The RMSF of residues present on the C helix and the loop between the C-β3 also showed considerably higher flexibility in comparison to WT FC (Figure S36).

The conserved E114 residue is proposed to provide a microenvironment in the active site of WT FC to promote the arginine residues to act as bases and initiate catalysis. The mutation E114Q resulted in a dramatic loss of enzyme activity (reduced by 84% relative to WT FC), but had no effect on SAM binding^12^. In this mutant there was increase in the RMSDs of the UP2 substrate (3.5 Å) in respect to the WT FC (2.3 Å), validating a role of E114 in substrate stabilization. The RMSF profile of UP2 in E114Q also showed higher fluctuations with respect to WT FC (Figure S37).

The M186 side chain interacts with both SAM and UP2, the mutation M186L affects both the enzyme activity (reduced by 84%) and strongly decreased SAM binding indicating an important role of M186 residue in stabilization of both SAM and UP2^12^. In the mutant M186l, there were no hydrophobic interactions (average distance 8.2 Å) observed between the side chain of L186 and methyl group of SAM. The backbone of L186 also moved away from the N3 of SAM and there was no hydrogen bonds observed due to increased distance of 7.2 Å between two groups (Figure S38). The L186 side chain no longer stabilizes the UP2 ring A and D with hydrogen bonds. The RMSD profile of UP2 in M186L also shows increased values in respect to WT FC (Figure S39) indicating the stabilizing role of M186 on the substrate UP2.

The mutations G189N and G189K significantly increased activity by 50% and 70% relative to WT FC^12^. In the WT FC, the G189 residue made no interactions with either SAM or UP2. The average distance of G189 from ring B propionate side chain is 7.7 Å. However in mutant G189N, N189 residue both side chain and the backbone participates in hydrogen bonds (average distance of 2.3 and 2.1 Å respectively) with the propionate side chain of ring B of UP2, which may indicate it stabilizes the substrate more than the WT (Figure S40). The stability of UP2 in the G189N mutant was also described by very stable and lower RMSD values in comparisons to WT FC (Figure S41). In the G189K mutant, the side chain of K189 made electrostatic interactions with the propionate side chain of ring B with an average distance of 4.0 Å (Figure S42). The backbone of K189 made hydrogen bonds with the propionate side chain of ring B of UP2. The RMSD analysis of UP2 in mutant G189K also showed more stability with respect to the WT FC (Figure S43).

The crystal structure^12^ described the involvement of K102 in maintaining the overall architecture of the active site by participating in a hydrogen bonding network with residues E115, G110, R100, Q81 and D50 (Figure S44, Figure S45). In WT FC MD simulation, K120 made electrostatic interactions with the side chain of E115 with an average distance of 3.7 Å. The backbone of K120 also made hydrogen bonds with R100. The side chain of R100 and Q81 stabilized the side chain of E115 by hydrogen bonding with an average distance of 3.2 and 2.9 Å respectively. Interestingly, the side chain of Q81 also made hydrogen bonds (average distance 3.4 Å) with the side chain of R111 during MD simulation and this interaction was not noted in the crystal structure^12^. The backbone of E115 made hydrogen bonds with the backbone of R111 residue; the E115 residue is located in vicinity of E114 and R111 residues which were demonstrated to be involved stabilization of UP2 in WTFC, the mutations *E114Q* and *R111K* reduce the enzyme activity.

The side chain of K102 in WT FC also made hydrogen bonds with the backbone of G110 which in turn stabilized the acetate side chain of ring D of UP2 by hydrogen bonding and also is present in vicinity of R111 residue. The mutation *K102A* completely negates the enzyme activity and reduces strongly the binding of SAM (Figure S46). The A102 in K102 mutant no longer made electrostatic interactions (average distance 8.3 Å) with the side chain of E115. This destabilization of the side chain of E115 in the vicinity of E114 and R111 also caused the Q81 (side chain) not to make hydrogen bonds with the side chain of R111 and backbone of E115 no longer made hydrogen bonds with backbone of R111. This destabilization of R111 side chain resulted in a lack of interactions (average distance 8.1 Å) with the acetate side chain of ring A of UP2. The side chain of E114 in K102A mutant made hydrogen bonds with the side chain of Q81 and shifted away from the active site. There were no strong interactions formed between the backbone of G110 and side chain of A102 as the average distance was 12.1 Å. As a result the backbone of G110 no longer stabilized the acetate side chain of ring D of UP2. The active site was dramatically perturbed due to a loss of many interactions in the K102 mutant, even though the K102 residue was not directly involved in binding with either UP2 or SAM. K102 stabilizes the active site in wild type NirE through a long range electrostatic effect.

The H161F mutant is described in the X-ray crystallographic study^12^ to reduce activity by ~48% but has no effect on the SAM binding. The aromatic ring of F161 in the H161F mutant made π-π stacking interactions with the ring system of UP2 (Figure S47, 48). However there were no hydrogen bonds formed by the side chain of F161 as compared to H161 in WT FC with the NH group of the pyrrole ring C of UP2. The local RMSF of residues in the vicinity of the F161 (±10 residues) in H161F also showed significant increase in respect to WT FC (Figure S51).

### Conformational Flexibility and Ligand Binding

The RMSD profile of the APO, ES and EC equilibrated around 12 ns and were stable throughout the simulation (Figure S50). However the RMSD trajectory of EC complex showed a slight shift in RMSD profile close to 30 ns in comparison to WT FC. It was evident from the simulation that binding of the substrate (UP2) had the greatest stabilizing effect on WT FC. The binding of both cofactor and substrate resulted in increased structural deviation in WT FC (3.2 Å) in contrast to APO (2.4 Å). The RMSFs of EC complex show high fluctuation relative to APO, ES and WT FC (Figure S51, Table S4). The loop between β3 and the D alpha helix (residues 70–80) and residue 163–179 between β6 and β7 which are mainly involved in stabilizing UP2 substrate including R51 and R111 in WT FC showed significantly higher fluctuations in EC. The data clearly indicates that the residues in the vicinity of the substrate UP2 showed high fluctuations in its absence in the EC complex and indicate their importance in substrate stabilization. The normalized distribution of center of mass distance between domain A and B in WT FC (monomer A) (Figure S52) showed an average increase of 29.7 Å with respect to APO (27.5), ES (28.1) and EC (27.7). This data clearly indicates that the two domains open up or adopt an extended conformation during the simulation, possibly to accommodate the cofactor (SAM) and large substrate UP2. The binding of cofactor and substrate also increased the solvent accessible surface area of WT FC in contrast to APO enzyme (Figure S53) which also points towards the idea of a more opened conformation of the enzyme when the substrate and cofactor binds.

### Dynamic Cross Correlated Motions

The cross correlation analysis allows the exploration between correlated and anti-correlated motions in proteins^17^. The N terminal region (residues 13–22) of the WT FC (Figure S54) showed correlated motion against the β4 and the loop between β4 and D α-helix (90–102 residues). The β4 sheet which is in close proximity to the cofactor SAM also showed correlated motion towards the residues 110 to 115 which belong to a loop between β4 and E α-helix and participles extensively in substrate binding in WT FC. The residues 37–47 of B α- helix and loop between B α-helix and β2 showed correlated motion against residue of β3 and loop region between β3 and D α-helix. The residues 37 to 47 also showed correlated motion against the residues 90 to 102 (loop between β4 and D α-helix). Interestingly the residues of monomer A (32–42) showed correlated motion towards residues of B α-helix and loop between B α-helix and β2 of monomer B. This loop contains residues which make interactions with the adenine ring of the cofactor SAM. The cross correlation motion map of APO showed a similar pattern of correlated motion compared to WT FC however the extent of correlated motion was decreased overall in particular in the region between β4 and D α-helix (90–102 residues) compared to the WT FC. There was also increased anti-correlation motion in APO in contrast to the WT FC (Figure S55). The greatest increase in anti-correlated motion was seen in the EC with respect to WT FC. The residues of the loop between β3 and D helix show anti-correlated motion towards residues of β7 and loop residues between I α-helix and β10. The residues of loop β7 and I α-helix make interactions with the substrate in the WT FC (Figure S56, S57). The ES complex showed extremely low anti-correlated motion and the profile was very similar to that of the WT FC, however the extent of the correlated motions was overall decreased (Figure S58). The cross correlation analysis indicates that complex molecular motions are involved in the binding of the substrate and cofactor and that their binding influences the pattern of the correlations.

The MD simulations provide important missing knowledge about how the flexibility and relaxation influence the structures of the ES complex, complementing the static picture obtained from X-ray crystallography. Furthermore we can use multiple structures of the ES complex from the MD trajectory to perform reaction mechanism studies using Combined Quantum Mechanics and Molecular Mechanics (QM/MM) methods. This will allow us to explore the effects of the conformational flexibility on key structures from the reaction path and on the activation energy of the reaction.

Our findings could be used as a basis for comparative modelling studies of other SAM-dependent uro’gen III methyltransferases (SUMTs), involved in cobalamin (CobA) and siroheme (CysG, SirA, UPM1, Met1p) biosynthesis. CobA, SirA, Met1p, like NirE are dimers and have only SUMT activity, whilst CysG is also a dimmer but carries out two independent enzyme activities. Molecular simulations can provide atomistic details, important for the explanation of key differences between these enzymes and open up new possibilities for the design of enzymes with these desired properties.

## Conclusions

By applying a large number of Atomistic Molecular Dynamics Simulations on different forms of the SAM-dependent Methyltransferase NirE (Full, complex, ES complex, EC complex, Apoenzyme and nine experimental mutants), we have provided an insight into the enzyme structure-function relationships which cannot be gained experimentally. We complemented the crystal structure information with information at atomistic levels about the impact of dynamics on enzyme structure and enzyme interactions with its substrate, UP2, and cofactor, SAM. We provided atomistic structural insight into the effects of the mutations of nine important residues in the binding and catalytic sites, explaining the experimentally measured effects on both binding and catalysis. The study asserts the importance of understanding protein dynamics in addition to the crystallographic, biochemical, and kinetic studies, thus providing a synergistic insight into our understanding of NirE’s structure-function relationships. The results provide a basis for further investigation of the enzyme mechanism using QM/MM methods, free energy of binding as well as applications in chemical biology and biotechnology.

## Methods

The coordinates of the wild type NirE were obtained from the Protein Data Bank (PDB)^18^ (PDB ID 2YBQ)^12^. The missing residues were added using PyMOL^19^ to the wild type NirE^12^. The methyl group was added to the cofactor molecule SAH using GaussView 5.0^20^. The resulting SAM molecule was used as a cofactor for the molecular dynamics simulation of NirE. There were nine experimental single amino acid mutants^12^ used in this study (Table S1). Mutant models were prepared using the What IF server^21^. The force field parameters for cofactor and the substrate were calculated using PRODRG^22^. The Molecular Dynamics simulations were performed using the Gromacs 4.5.5 package^23^,^24^ with the GROMOS96 43a1 force field^25^. Hydrogen atoms were added to the protein molecule using pdb2gmx utility in Gromacs. The energy minimization in vacuum were performed initially on the protein structure using the steepest descent method^26^ until the maximum force was smaller than 100 KJ/mol^−1^/nm^−2^. The protein molecule was placed in a cubic box with a cut-off distance of at least 1.0 nm between the protein and the box boundaries using the editconf command. The minimized protein structure was then solvated using the Single Point Charge^27^ (SPC) water model and periodic boundary conditions were then applied to treat all the parts of the system equally both at its interior and edges. The system was neutralized by adding an appropriate number of Na^+^ and Cl^-^ ions to the various mutants and the wild type enzyme. This was followed by energy minimization of the system using first the steepest descent method followed by a conjugate gradient algorithm until the maximum force was smaller than 100 KJ/mol^−1^/nm^−2^. The energy minimized structure was then subjected to position restrain dynamics for 50 ps. The simulation was performed in NVT ensemble^28^ (constant Number (N) of particles, Volume (V), and Temperature (T)) at constant temperature of 300 K with a time step of 0.002 ps. The productive 50ns MD was carried out using NPT (constant number of particles (N), system pressure (P) and temperature (T)) ensemble at constant temperature of 300 K and the initial velocities for MD simulation were drawn from Maxwell velocity distribution at 300 K. The MD was performed with an integration time step of 0.002 ps. The Particle Mesh Ewald (PME) method^29^ was used for electrostatic interactions, the van der Waals interactions were treated using a Lennard-Jones potential. The cut-off distance for van der Waals interaction was set to at 1.0 nm. The coordinates were saved after every 20 ps from multiple MD trajectories. The analyses of the trajectories obtained from the simulations were performed using tools from the Gromacs software package and VMD^30^. The visualization of MD trajectories and the structures was performed using VMD software^30^. The Bio3D package^31^ in R^32^ was used to perform dynamic cross correlation Analysis. The positive value represents the correlated motion and the negative values represent the anti-correlated motion. The R^31^ and Xmgrace were used to prepare plots and analysed data in this study.

For better clarity we provide the enzyme activity of the mutant forms as percentages from enzyme activity of the wild type^12^. We accepted the enzyme activity of the wild type which is 10.5 to be 100% and recalculated the respective values for each from the mutants^12^. For SAM binding activity we provide only qualitative indication as in ref. 12.

## Additional Information

**How to cite this article**: Singh, W. *et al.* Conformational Dynamics, Ligand Binding and Effects of Mutations in NirE an S-Adenosyl-L-Methionine Dependent Methyltransferase. *Sci. Rep.* **6**, 20107; doi: 10.1038/srep20107 (2016).

## Supplementary Material

Supplementary Information

## Figures and Tables

**Figure 1:**
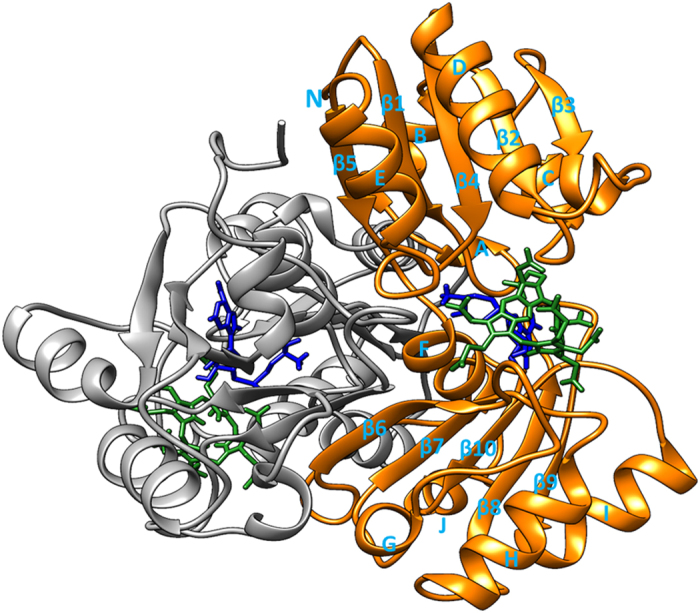
The 3D X-ray crystal structure (PDB code: 2YBQ)^12^ of NirE enzyme complexed with Uro’gen III substrate and SAH cofactor using UCSF Chimera^32^. (A) The homodimer NirE enzyme is represented in monomer (A,B) in orange and grey new cartoon representation respectively. The spent methyl donor product SAH and substrate Uro’gen III are shown in sticks representation in dark green and blue colours respectively. The loop of monomer (B) interacts with the active site of monomer (A) especially with substrate UP2 and vice versa.
