# Supplementary material for: Conformational Dynamics, Ligand Binding and Effects of Mutations in NirE an S-Adenosyl-L-Methionine Dependent Methyltransferase
Source: Sci Rep. 2016 Jan 29;6:20107. doi: 10.1038/srep20107 (PMC4731766; doi:10.1038/srep20107)
Supplement: Supplementary Information [file srep20107-s1.pdf]

Conformational Dynamics, Ligand Binding and Effects of Mutations in NirE an S-Adenosyl-L-Methionine Dependent Methyltransferase

Warispreet Singh[1‡], Tatyana G Karabencheva-Christova[1‡*], Gary W Black[1], Jon Ainsley[1], Lynn Dover[1] and Christo Z Christov[1*]

[1]Department of Applied Sciences, Faculty of Health and Life Sciences, Northumbria University, Newcastle upon Tyne, NE1 8ST, United Kingdom

Correspondence and request for materials should be addressed to C.Z (*christo.christov@northumbria.ac.uk) or T.K-C (*tatyana.karabencheva-christova@northumbria.ac.uk)

‡ Equal contributions

Supplementary Information

Table S1 The setup of the NirE enzyme and the mutants for Molecular Dynamics simulations

S.No	MD-setup	Occurrence	Timescale (ns)
1	Wt NirE (WT FC)	Chain A + B + UP2 + SAH	50
2	ES	Chain A + B + UP2	50
3	EC	Chain A + B + SAH	50
4	APO	Chain A + B	50
5	Deprotonated R111	β4/E helix	50
6	Deprotonated R*149	F helix/β6 of chain B interacts with chain A	50
7	Deprotonated R111, R*149	β4/E helix, F helix / β6	50
8	E114Q	E helix	50
9	G189K	H helix	50
10	G189N	H helix	50
11	H161F	β6/G helix	50
12	M186I	β7/H helix	50
13	R111K	β4/E helix	50
14	R*149K	F helix / β6	50
15	R51K	β2/C helix	50
16	K102A	β4	50

Table S2 The multiple run trajectory of the wild type NirE (WTFC) for 50 ns.

Name	Mean (Å)	S.D* (Å)	S.E.M* (Å)
Run 1	3.2	0.41	0.0083
Run 2	2.9	0.27	0.0055
Run 3	2.6	0.28	0.0056
Averaged all	2.9	0.30	0.0064

* S.E.M Standard Error of Mean, S.D Standard Deviation

Table S3: The average and standard deviation of RMSD of WT FC and the mutants along with RMSF analysis

Name	Mean (Å)	S.D(Å)	RMSF > 1.1 Å (%)	RMSF < 1.1 Å (%)
WT FC	3.2	0.41	47	53
APO	2.4	0.15	31	69
ES	2.4	0.20	25	75
EC	2.9	0.42	48	52
E114Q	2.4	0.18	29	71
G189K	2.9	0.34	41	59
G189N	2.6	0.22	26	74
H161F	2.7	0.27	27	73
M186L	2.9	0.30	40	60
R*149K	2.7	0.29	42	58
R51K	2.6	0.22	40	60
R111K	3.1	0.43	38	62
R111K –Deprotonated	2.7	0.26	40	60
R*149K – Deprotonated	2.4	0.26	45	55
K102A	2.7	0.25	37	63
R111, R*149K – Deprotonated	2.5	0.15	30	70

R*149 represents the residue of monomer B interacting with residue of monomer A

Table S4: Local structural RMSF of WTFC and mutants of NirE, the region is chosen to increase or decrease if there is an average change in RMSF of > 0.3 Å in at least 50% of its residue

Name	Increased		Decreased	
	Residue span	Name	Residue span	Name
R*149K	55-63, 75-78, 160-170, 188-205, 228-233	C C-β3, β3-D, β6-G, H H-β8, I	175-178, 218-221	G-β7, β8-β9
H161F	73-78, 162-170	β3-D, β6-G	26-36,134-143,188-194,226-237	β1-A A-B, F F-β6, H, β9-I
M181L	55-63, **73-78,** 82-92, **162-171**	C C-β3, β3-D, D, β6-G,	26-36, 134-150, 173-176	β1-A A-B, F F-β6, G
G189N	75-77, 162-168	β3-D, β6-G	25-37, 148-151, 190-193, 232-238	β1-A A-B, F-β6, H, I
G189K	55-59, 72-90, 110-125,188-194	C, β3-D D, E E-β5, H	25-35,134-140,172-176	β1-A A-B , F, G
R111K	53-64, **79-85**, 110-117, **163-168**	C C-β3, β3-D D, E, β6-G	27-29, 133-144, 235-237	β1-A, F F-β6, I-β10
E114Q	72-78, 80-87, 163-171	β3-D, D, β6-G	26-39, 133-144, 172-175, 188-193, 231-238	A B, F F-β6, G, H, I-β10
R51K	50-55**, 74-87,** 103-115, **161-168**	β2-C, β3-D D, β4-E E, β6-G	25-34, 143-150, 173-177,	β1-A A-B, F-β6, G
K102A	52-55, 70-85, 110-113, 160-167,231-239	β2-C, β3-D D, β4-E, β6-G, I- β10	27-37, 171-180	β1-A, G G- β7
ES	71-86, 110-120	B3-D D, E	25-36, 133-145, 169-179, 196-206, 228-237	β1-A A-B, F F-β6, G G-β7 , H H-β8, I-β10
EC	51-61, 73-86, 109-115, 133-136, 162-173, 190-201	β2-C C-β3, β3-D D, E, F, β6-G, H	231-237	I-β10
APO	71-73, 163-168, 187-203	β3-D, β6-G, β7-H	32-35, 121-124,148-152, 174-177	A-B, E-β5, F-β6, G

* Regions underlined are the loops between the alpha helix and beta sheets.

Table S5 The hydrogen bonding* Interactions of substrate (UP2) and cofactor (SAM) with protein in WTFC

Donor	Acceptor	Distance (Å)	Probability (%)	Minimized Crystal structure
D105(bb)	SAM ($NH3^+$)	3.2	32.7	2.8
D105(sc)	SAM ($NH3^+$)	3.5 (30 ns)	21.5	5.5
I108(bb)	SAM ($NH3^+$)	2.9	76.9	2.8
Y185(sc)	SAM(COO^-)	2.2	85.6	3.5
T133(sc)	SAM(COO^-)	2.9	80.0	2.6
A134 (bb)	Y185 (sc)	2.8	32.4	3.6
SAM (N6)	C138 (sc)	3.4	26.2	4.0
SAM(N6)	P242 (bb)	3.3	3.0	5.0
SAM(N6)	V212(bb)	3.5	29	6.2
SAHM(N6)	Q214(bb)	3.6	45.7	4.5
Q217 (sc)	SAM (N7)	3.5	71.4	4.6
M186 (bb)	SAM (N3)	2.9	36.3	6.4
Ring C (NH)	H161(bb)	2.8	12	3.5
Q163 (sc)	Ring D Propionate (sc)	3.2	44.0	4.1
R111 (bb)	Ring D acetate (sc)	2.4	39	3.2
G110 (bb)	Ring D acetate (sc)	2.5	70	2.7

*Hydrogen bond criteria: Angle > 120° and donner acceptor distance < 3.5 Å
Sc = side chain, bb = backbone, $NH3^+$ (amino group), COO^- (Carboxylate group)

Table S6 The important interactions observed in WTFC in context of cofactor SAM and substrate UP2

Side chain	Side chain	Distance (Å) in md	Crystal structure
ring A *	Methyl	6.0	5.9
ring B *	Methyl	6.3	5.8
Methyl (SAM)	M186 (sc)	4.3	3.9
Methyl (SAM)	Y185	4.2	5.0
Methyl (SAM)	F109	4.0	3.8
P242 (sc)	SAM (adenine)	3.8	3.7
A134 (sc)	SAM (adenine)	3.6	3.6
Y185 (sc)	SAM (adenine)	3.6	3.9

L52 (sc)	SAM (CAI)	4.1	4.7
R111 (sc)	R*149 (sc)	8.9	3.6
R51 (sc-ali)	Ring A Propionate (ali)	4.1	5.7
ring A propionate (sc)	Sulphur	3.1	5.9
R111 (sc)	Ring A Acetate (sc)	4.3	4.8
D105 (sc)	$NH3^{+}$ (SAH)	4.0	5.6
H161(sc ali)	Ring C acetate (sc)	4.4	3.7
Q163 (sc ali)	Ring C Propionate (sc) ali	3.9	5.5
T159 (sc ali)	Ring D Propionate (sc) ali	4.0	5.1
L162 (sc ali)	Ring D Propionate (sc) ali	4.4	5.3

Ali = aliphatic side chain of amino acids, Cofactor (SAM)

Table S7 The average and multiple runs of R*149 deprotonated

Name	Mean (Å)	S.D (Å)	R*149 (sc) – C20 UP2
Run 1	2.4	0.25	3.5
Run 2	2.8	0.27	4.2
Averaged all	2.6	0.24	3.8

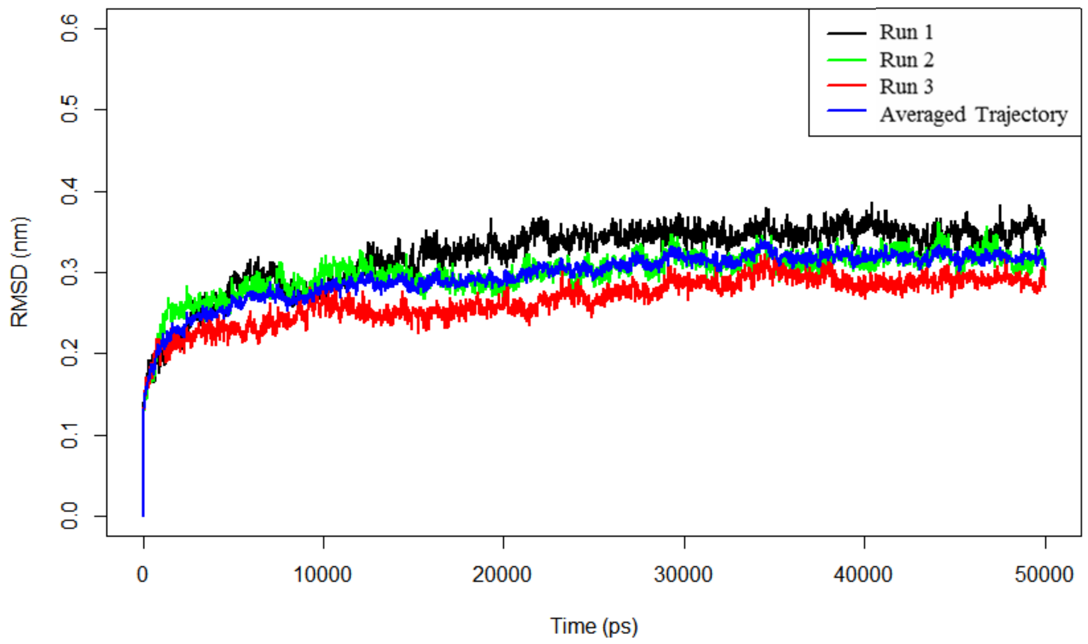

Figure S1 The multiple runs of the wild type NirE (WTFC) enzyme for 50 ns. The average trajectory of all the runs is represented in blue colour

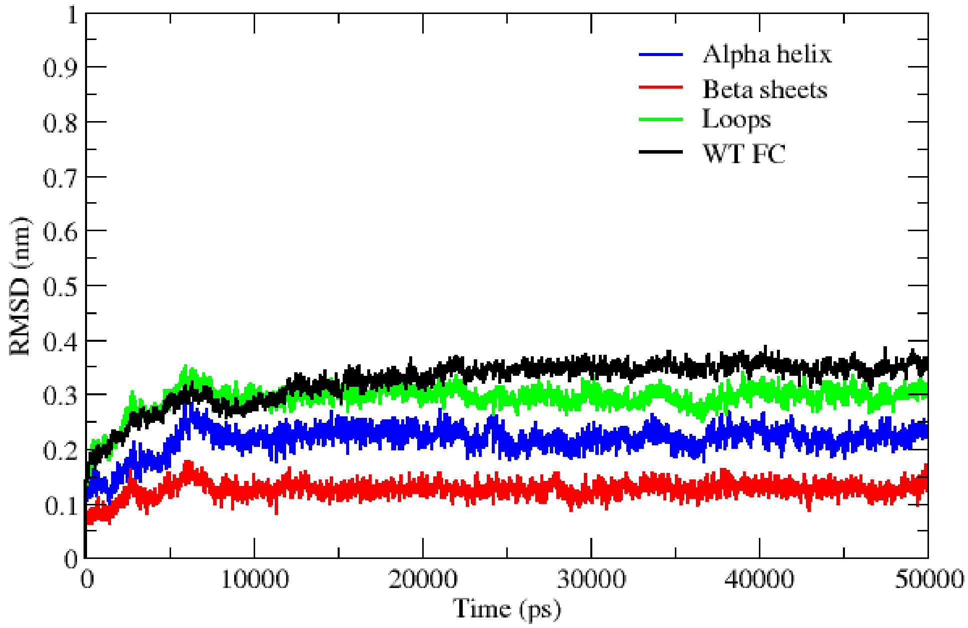

Figure S2 The RMSD decomposition of WT FC which include alpha helix beta sheets and loops for 50ns using C α atom

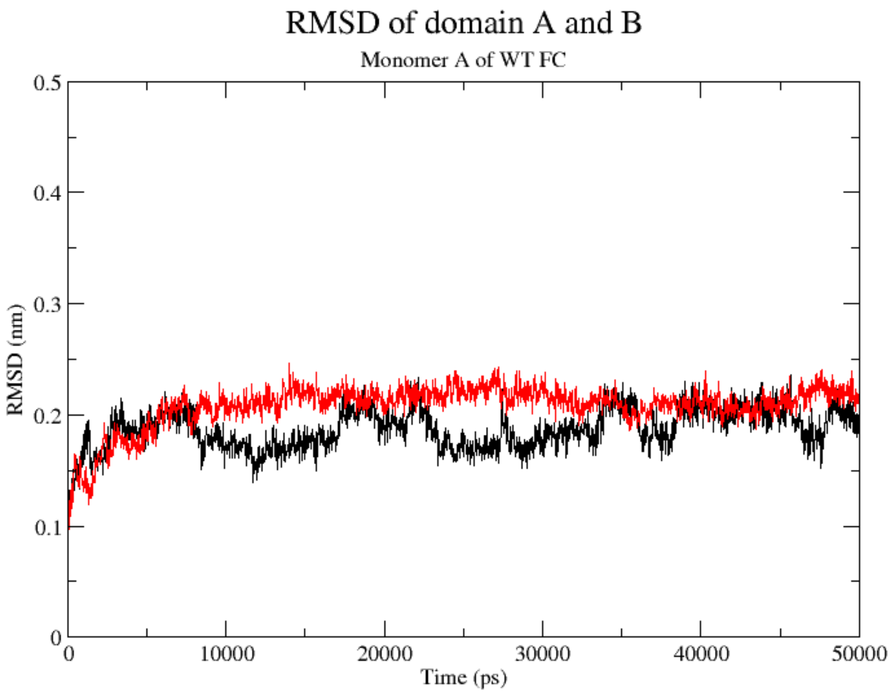

Figure S3 The RMSD of domain A and B of monomer A of WTFC for 50 ns.

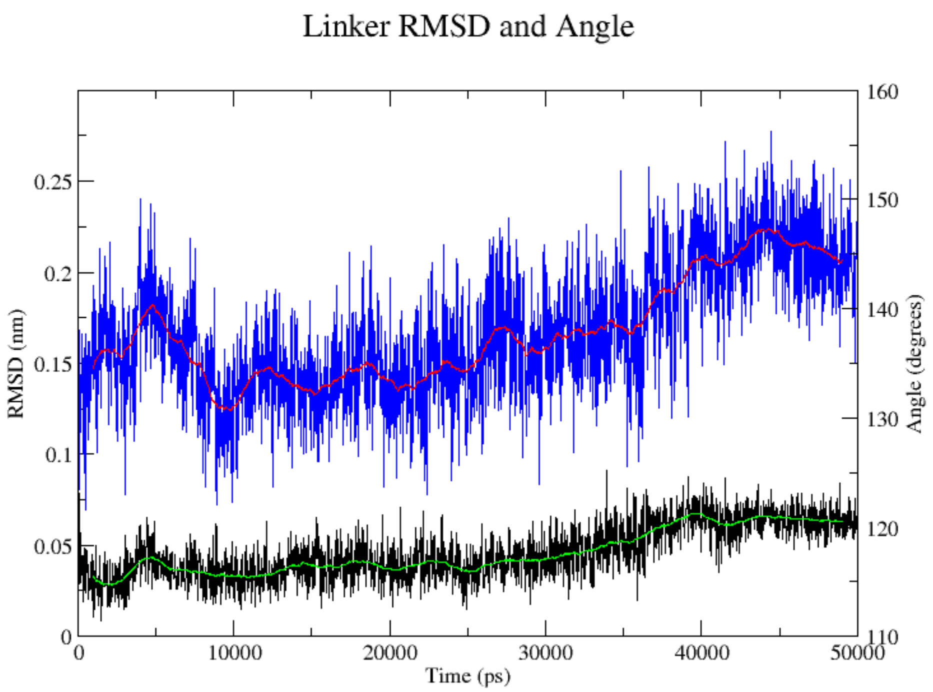

Figure S4 The RMSD of the linker region of WTFC and angle of the linker region residues (Cα of 129, 132 and 134) as function of time for 50 ns.

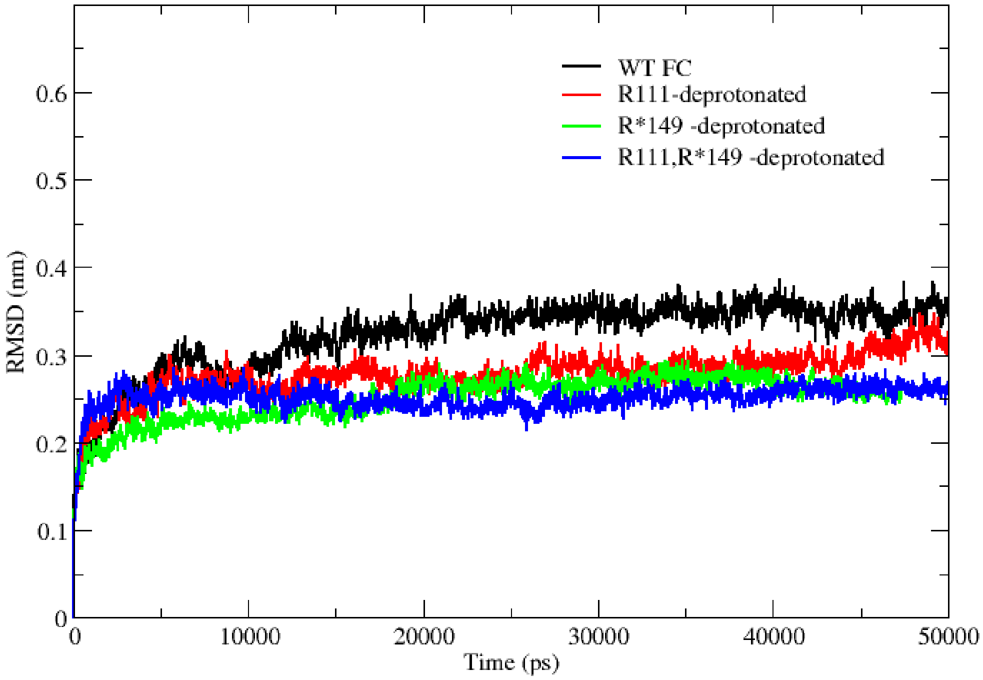

Table S5 The RMSD of the deprotonated arginine residue of the NirE enzyme for 50 ns.

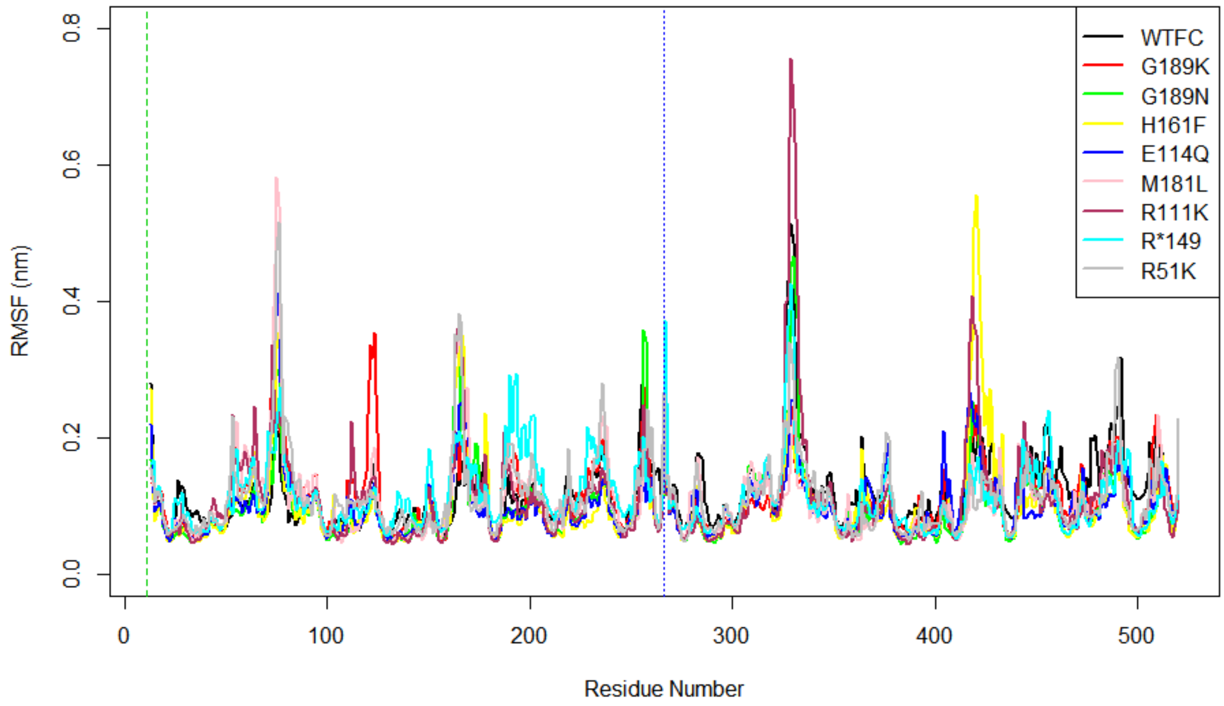

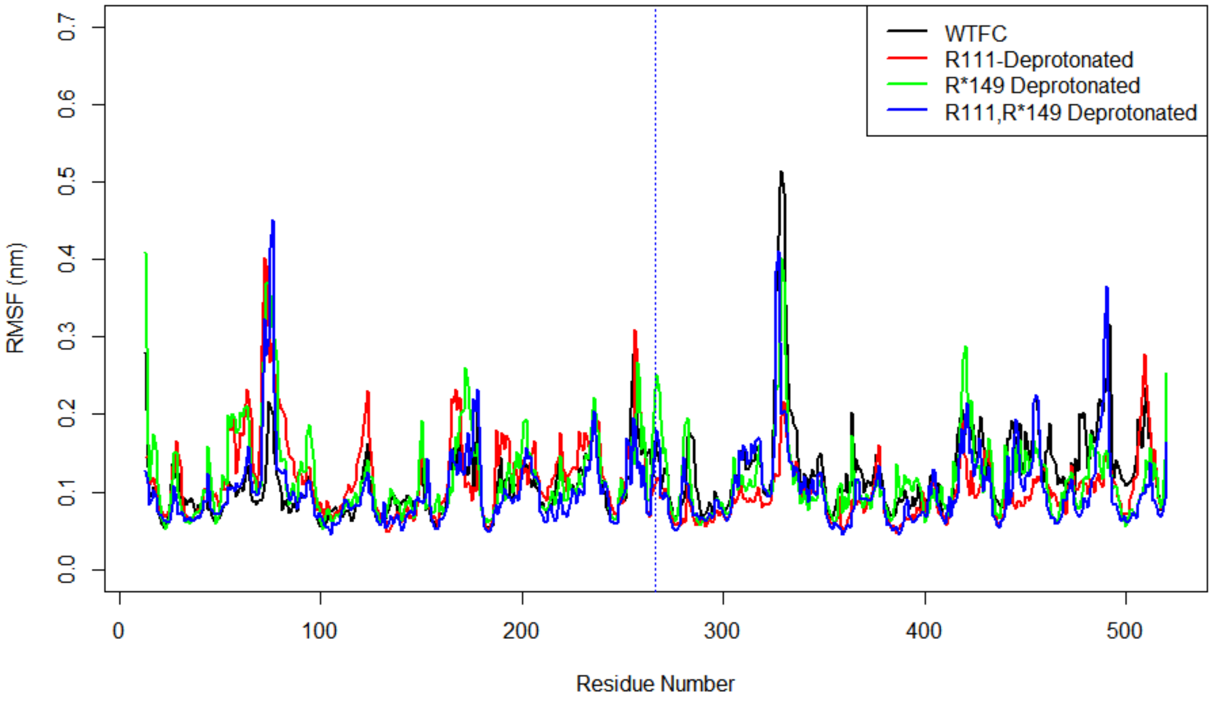

Table S6 The RMSF of the WTFC and the mutants for both monomer A and monomer B. The blue line separates the monomer A and monomer B. The green line in the start of the graph show that the residue starts according to the canonical naming in the crystal structure from residue number 13.

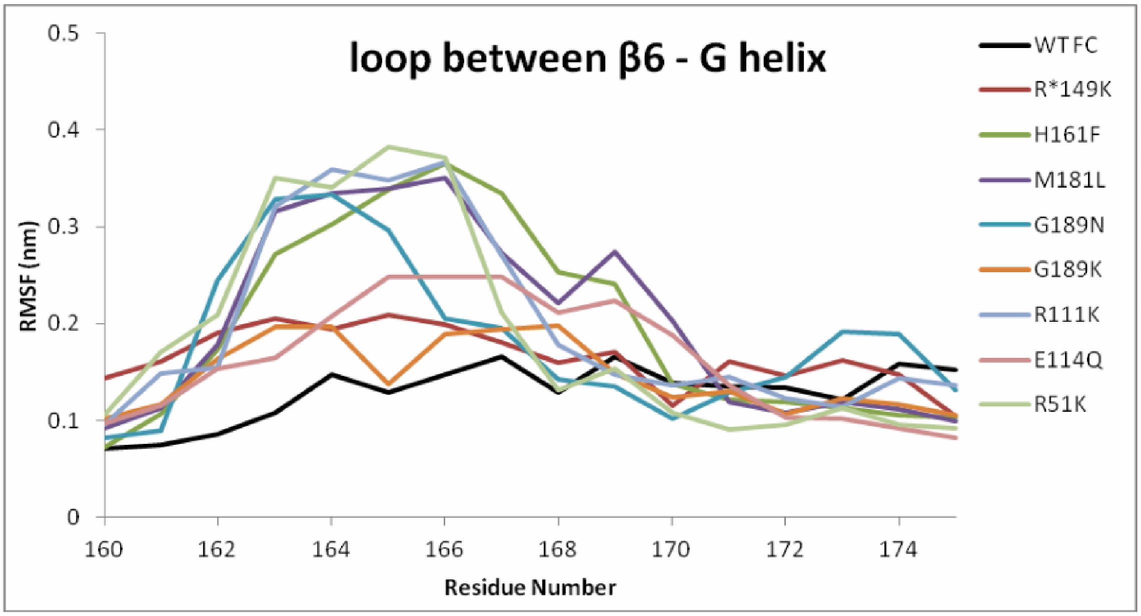

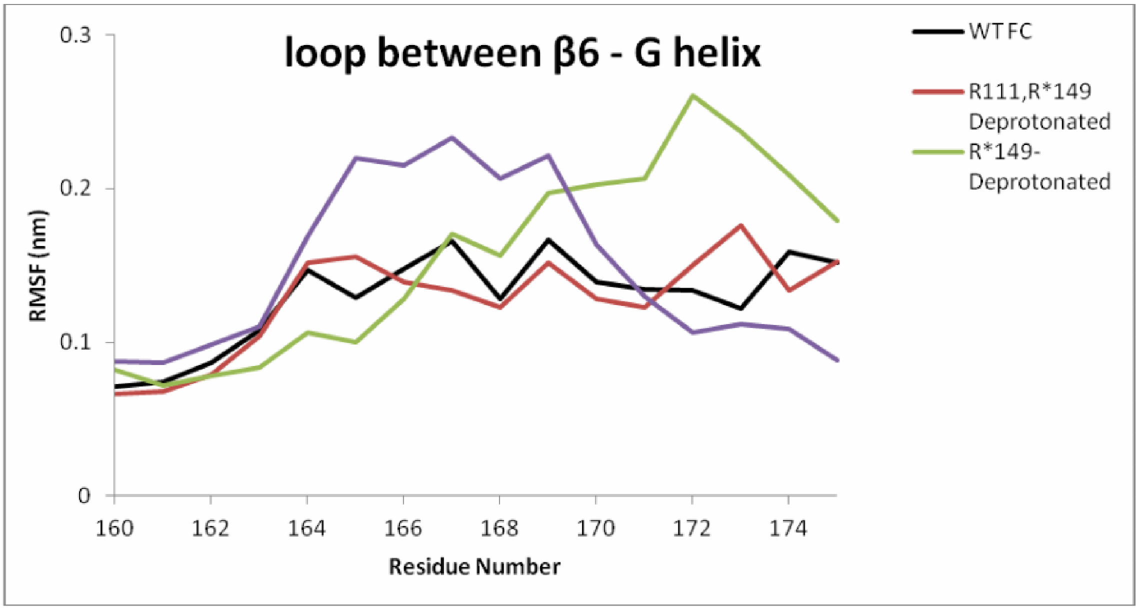

Figure S7 The RMSF of the mutants and WT FC of residues between loop β6-G helix

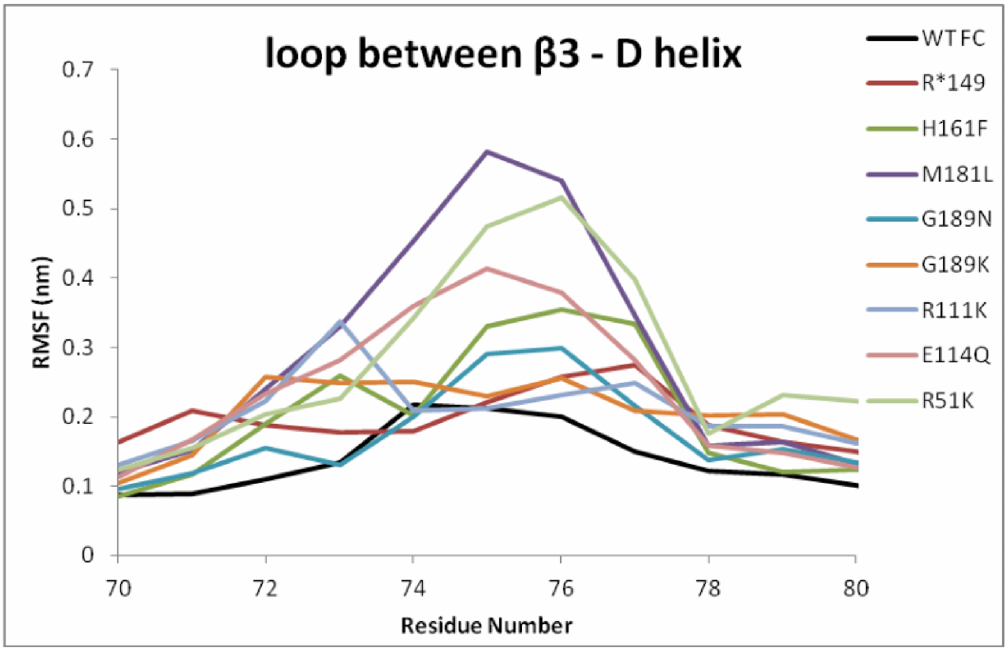

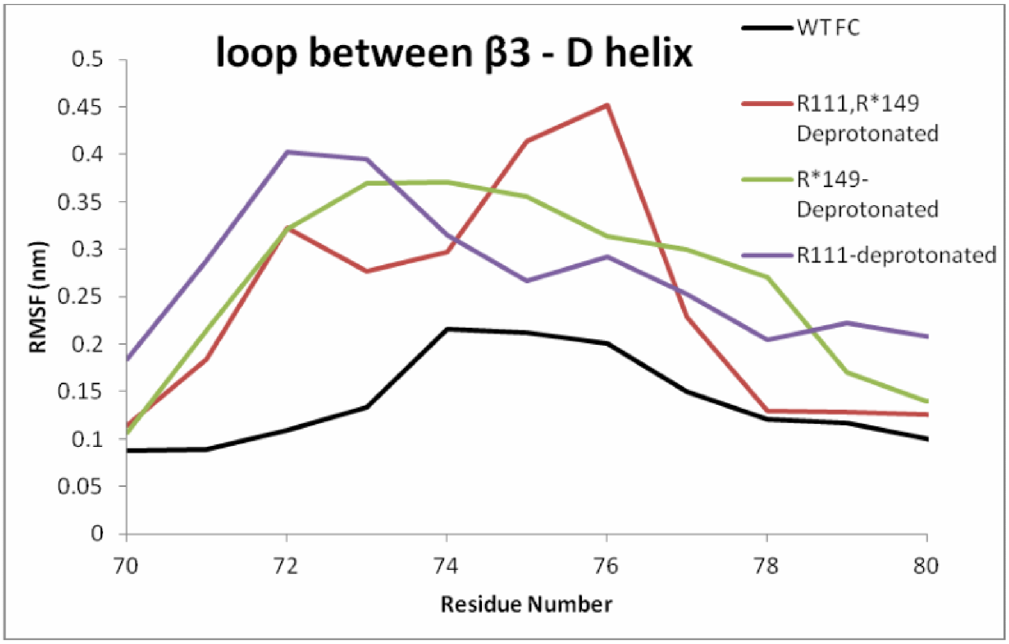

Figure S8 The RMSF of the mutants and WT FC of residue between β3- D

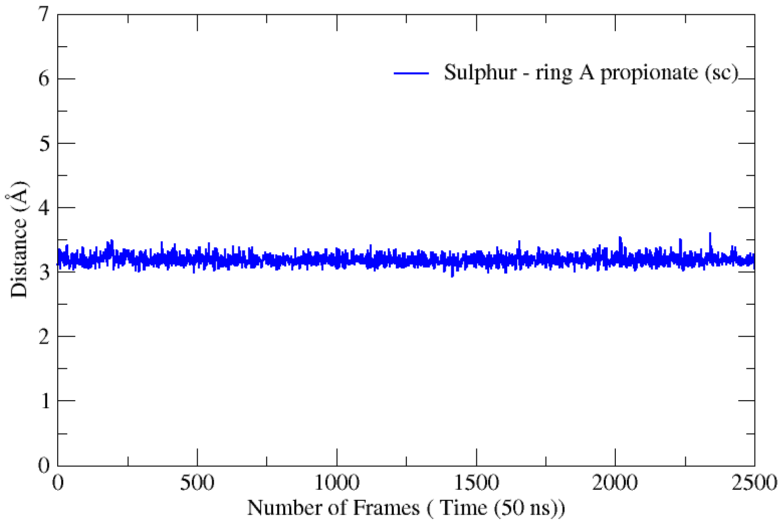

Figure S9 The sulphur atom of cofactor (SAM) electrostatic interactions with the propionate side chain of ring A of the UP2

A

Poteintial methyl transfer sites on substrate

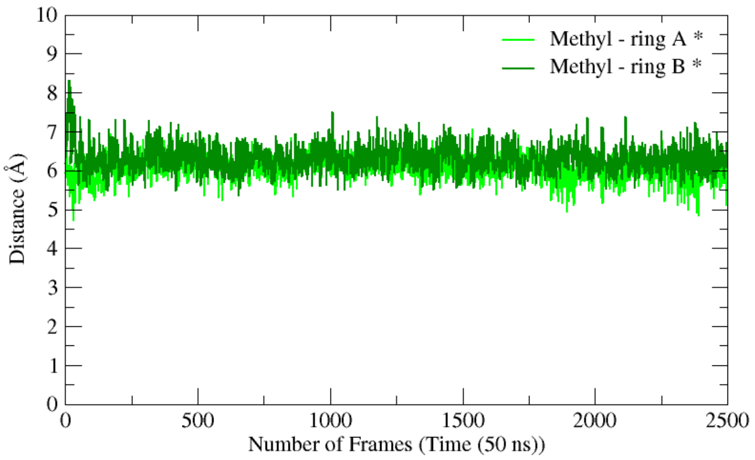

B

Angle between Sulphur - Methyl and Potential site on UP2

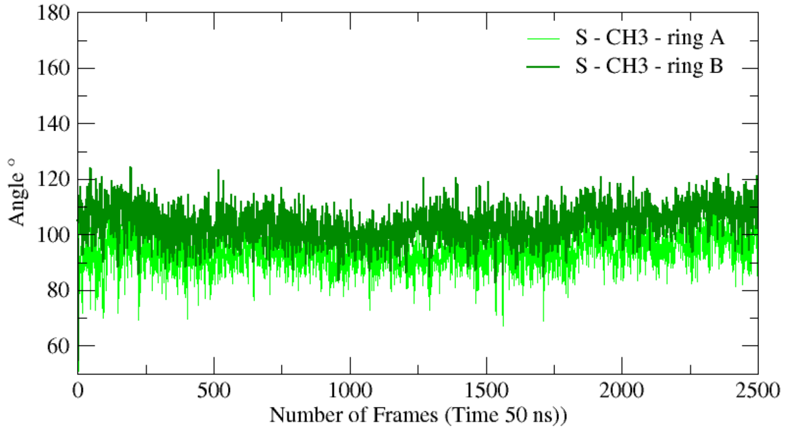

C

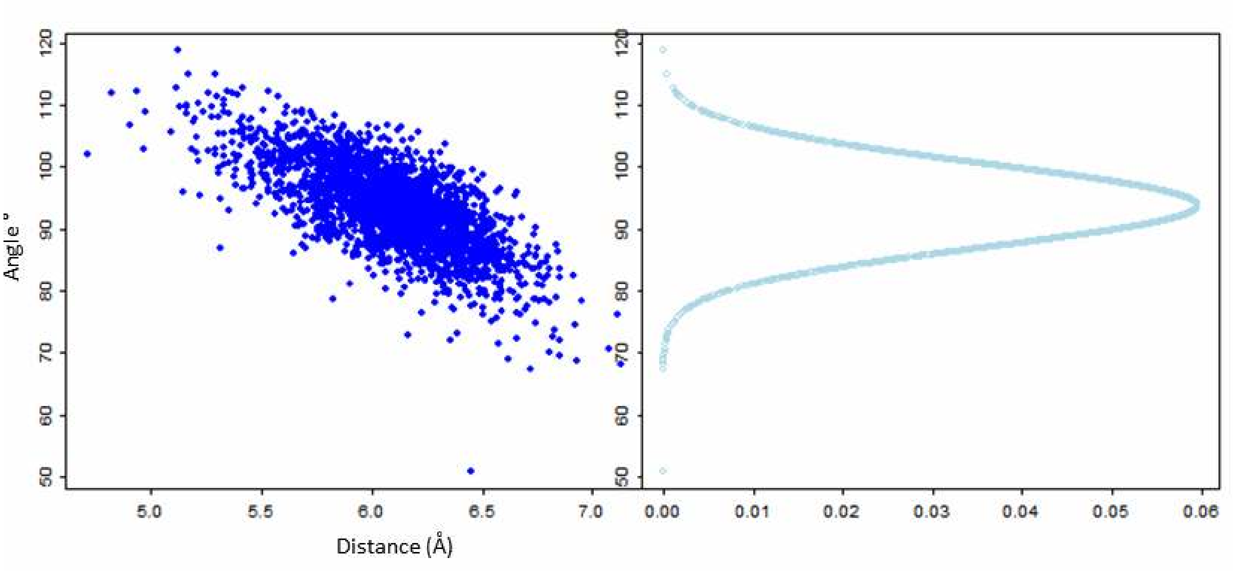

Figure S10 The methyl distance and angle between Sulphur, methyl group and methyl acceptor sties for the nire wild type for 50 ns simulation. (A) The methyl distance from the cofactor to its potential transfer site on the substrate , (B) The measurement of the angle between the sulphur, methyl and potential methyl acceptor site on the substrate, (C) The distribution of distance and angle

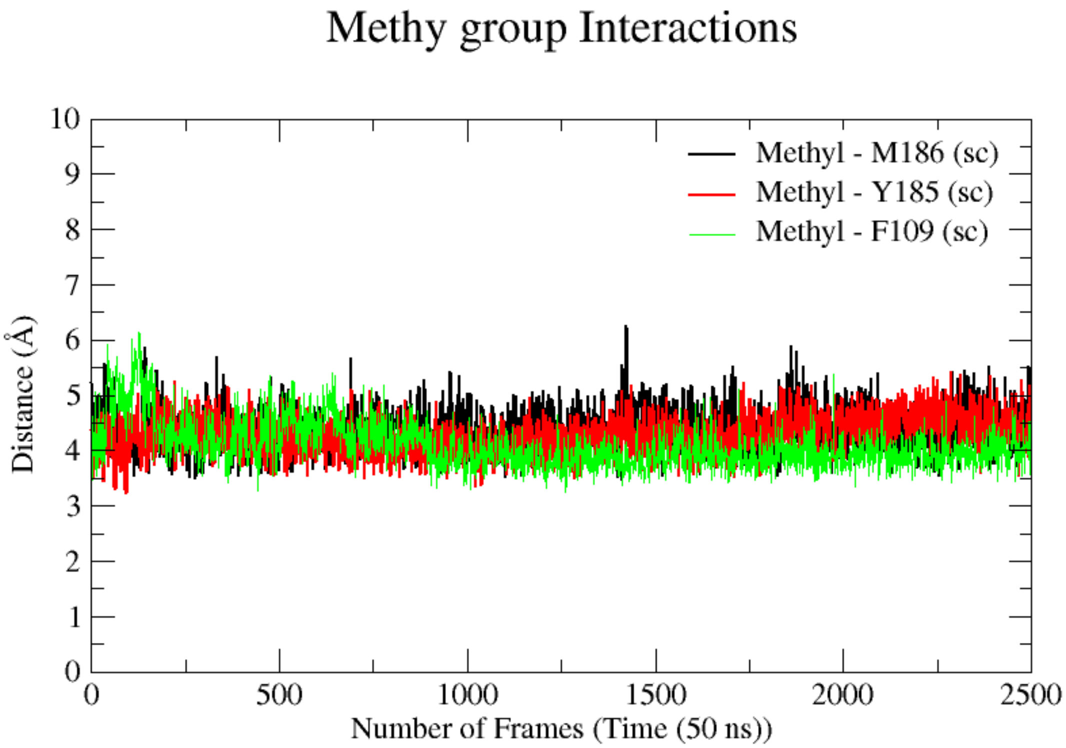

Figure S11 The interaction of newly added methyl group of cofactor SAM with the WTFC residues.

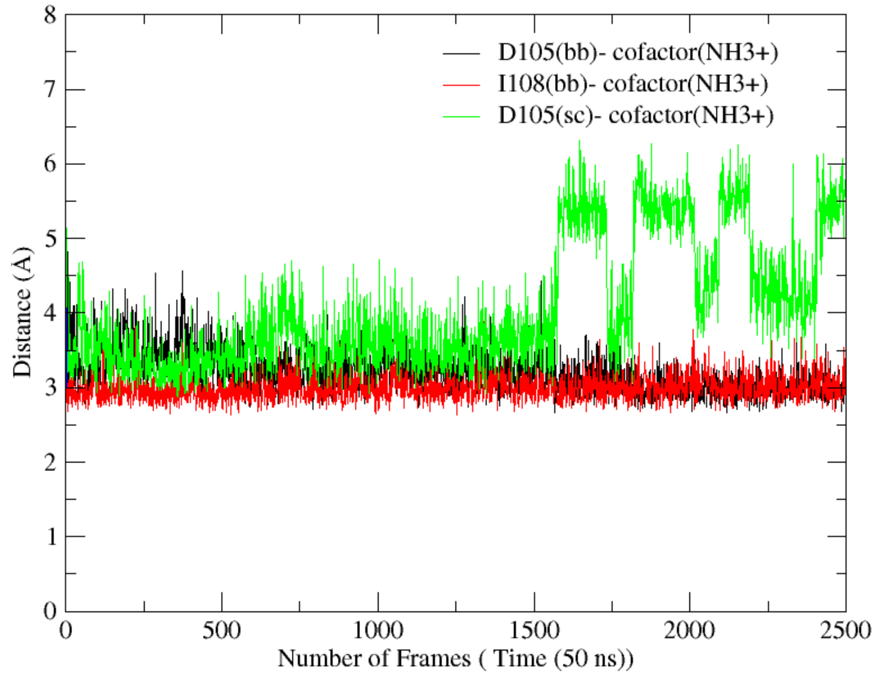

Figure S12 The interactions of WTFC residues with the N terminal group (NH^{3+}) of the cofactor SAM

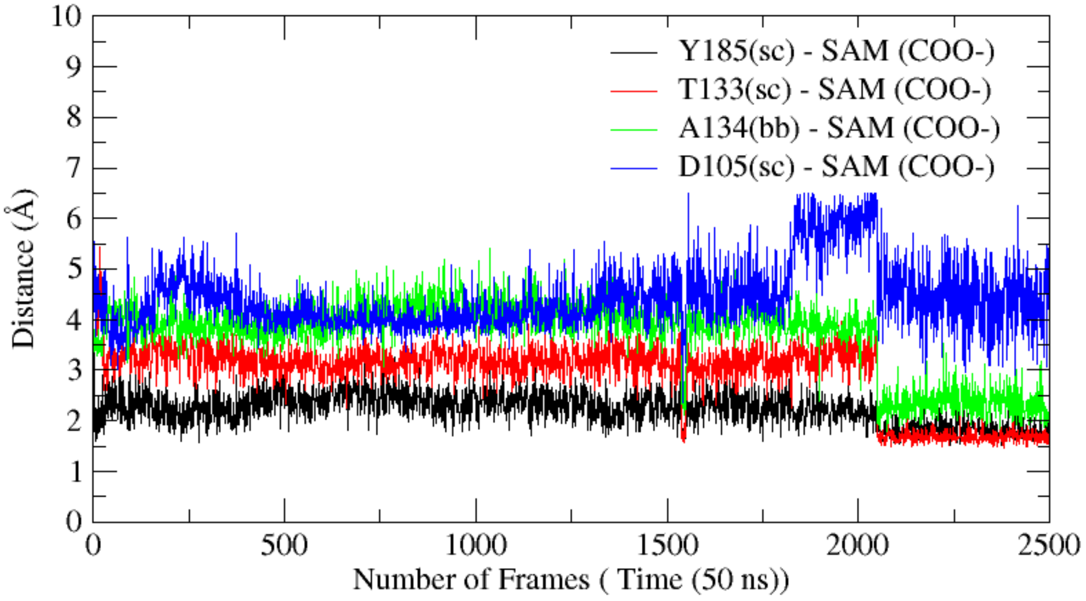

Figure S13 The interaction of the carboxylic group of the cofactor SAM with WTFC residues

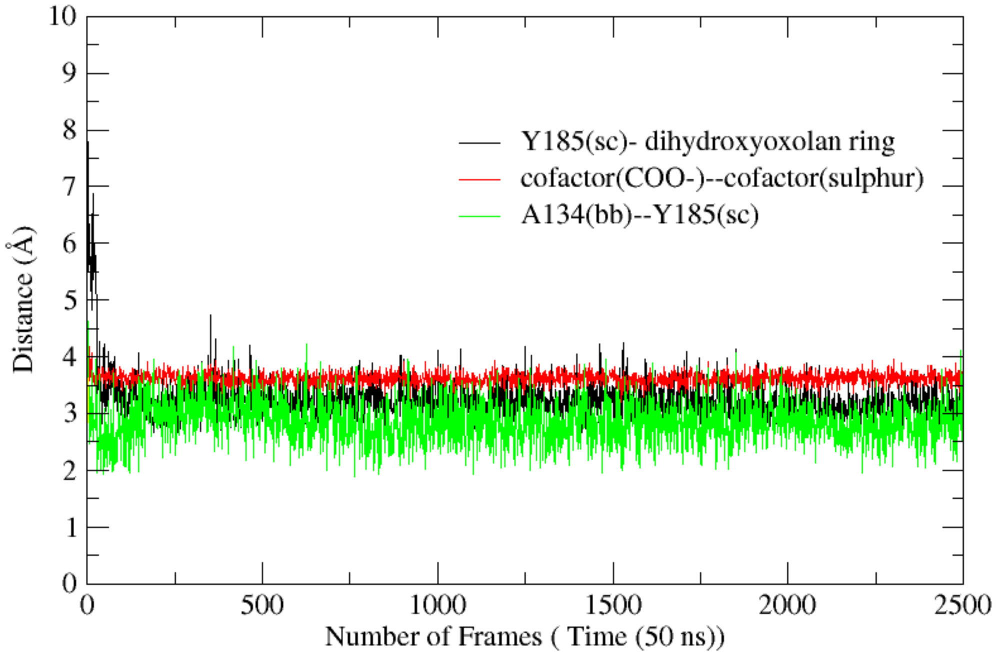

Figure S14 The interactions of the protein resdiue (WTFC) with the carboxylic group of the cofactor SAM.

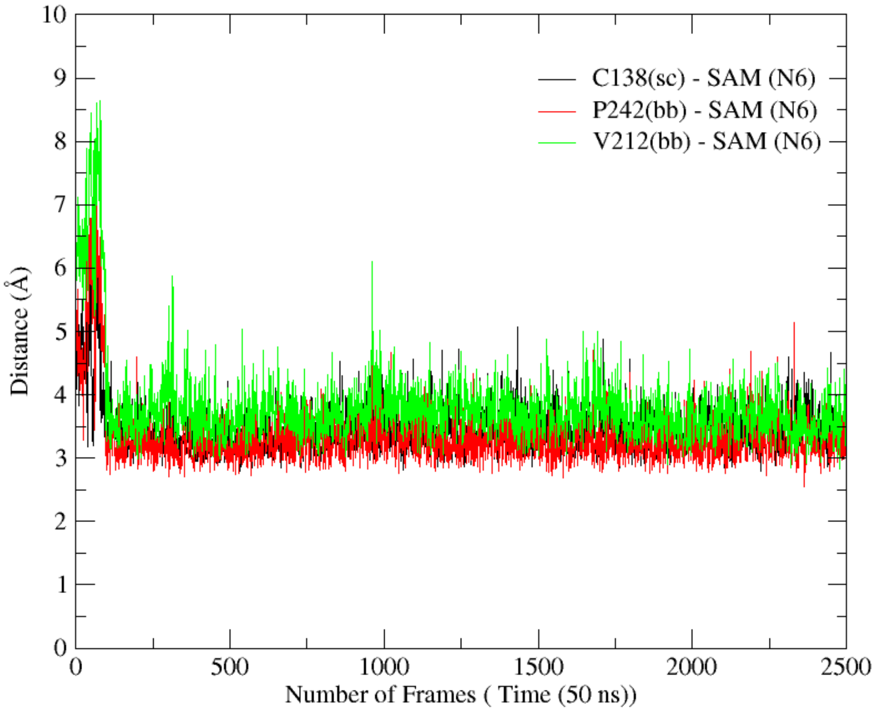

Figure S15 The adenine ring interactions of cofactor SAM with WTFC residues

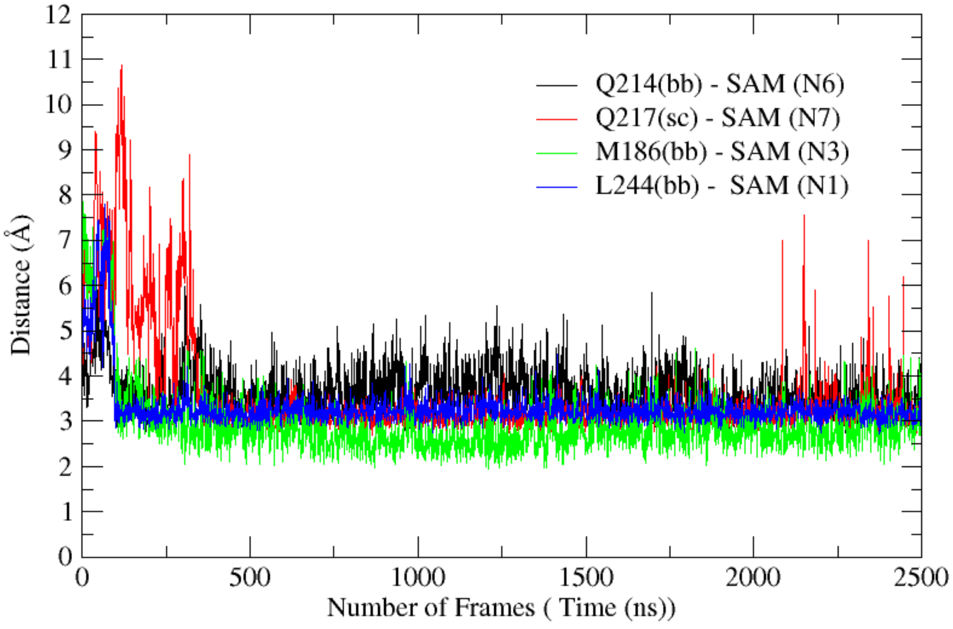

Figure S16 The adenine ring interactions of cofactor SAM with WTFC residues

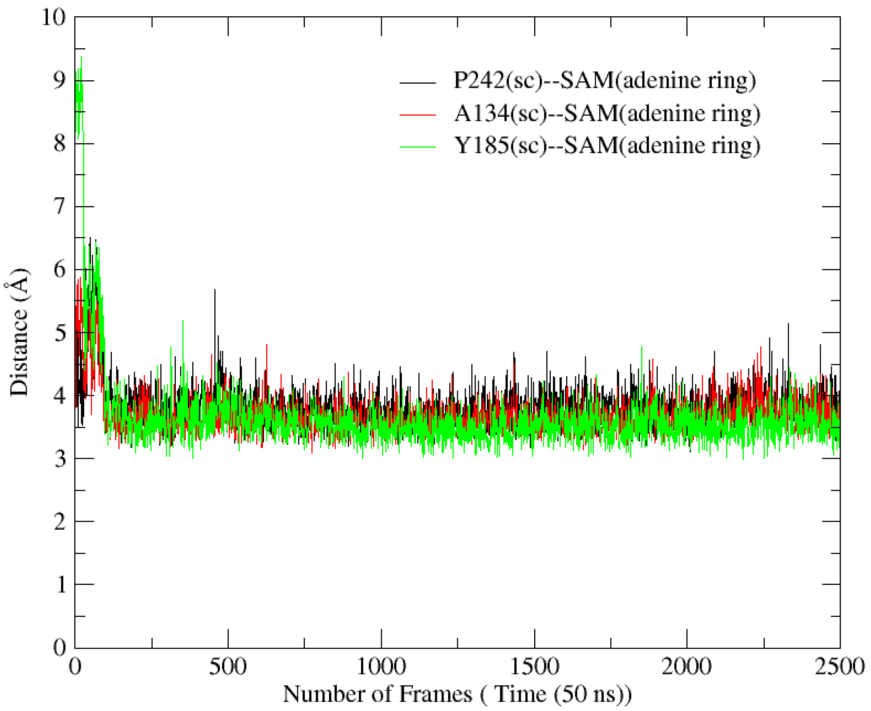

Figure S17 The adenine ring of the cofactor SAM and the hydrophobic interaction it makes with WTFC residues

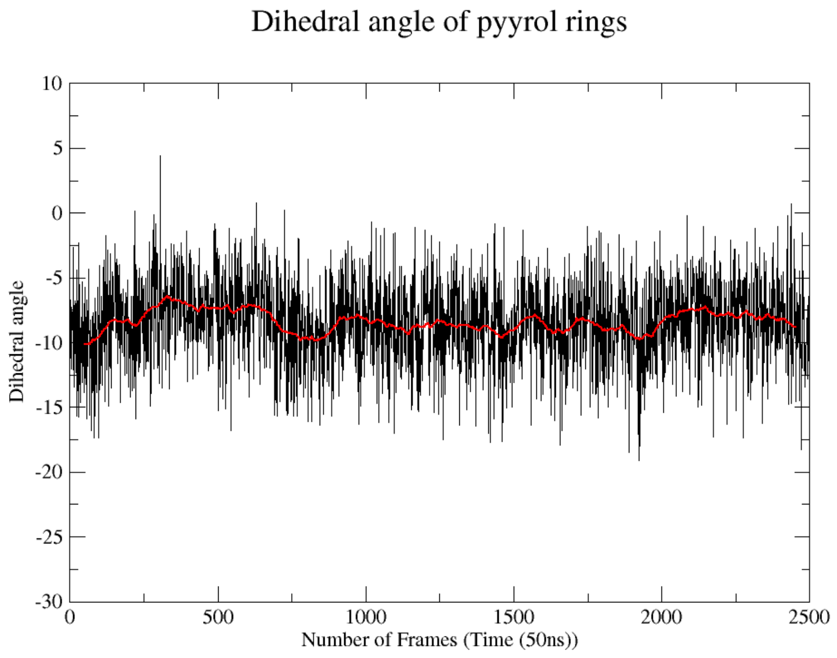

Figure S18 The dihedral angle of four pyyrole ring of the substrate UP2 in WTFC

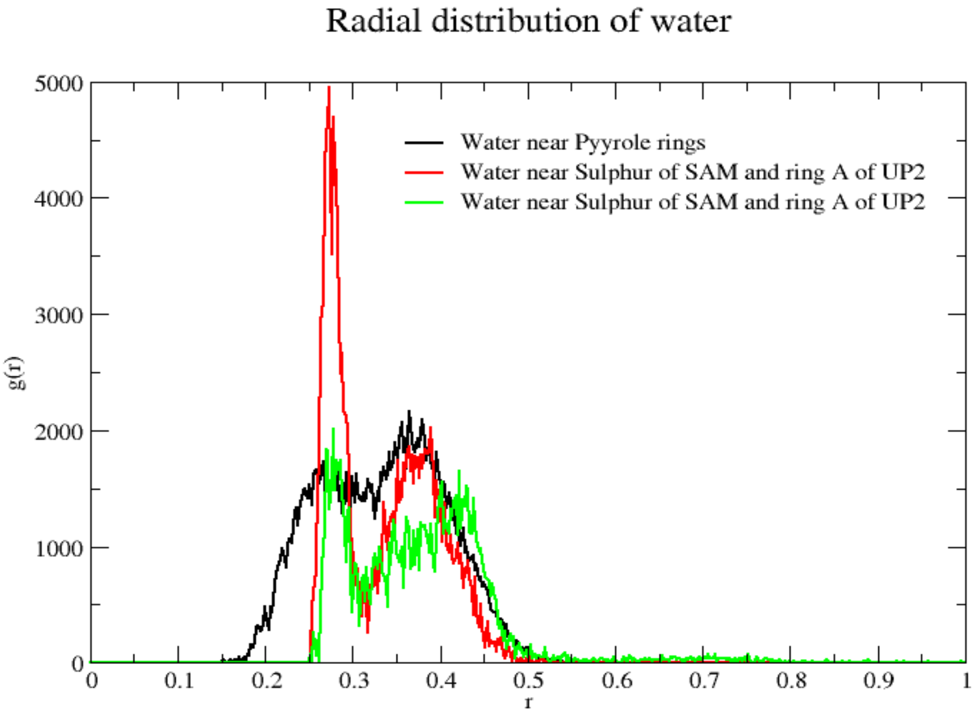

Figure S19 The radial distribution of solvent molecules near the UP2 and SAM of WT FC

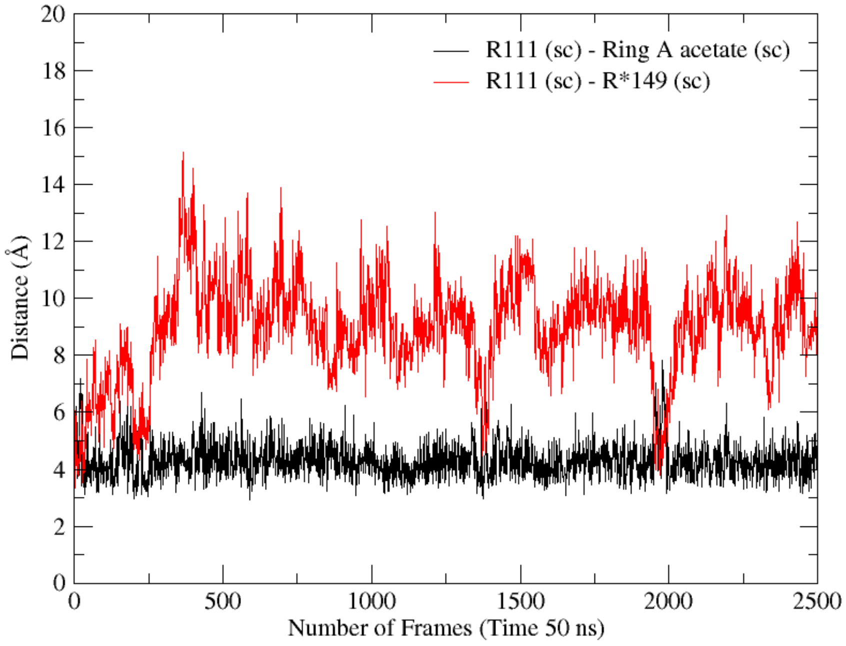

Figure S20 The interactions of the ring A acetate side chain with the residues of WTFC. The side chain of R111 distance with sidechain of R*149 of WTFC for 50 ns simulation

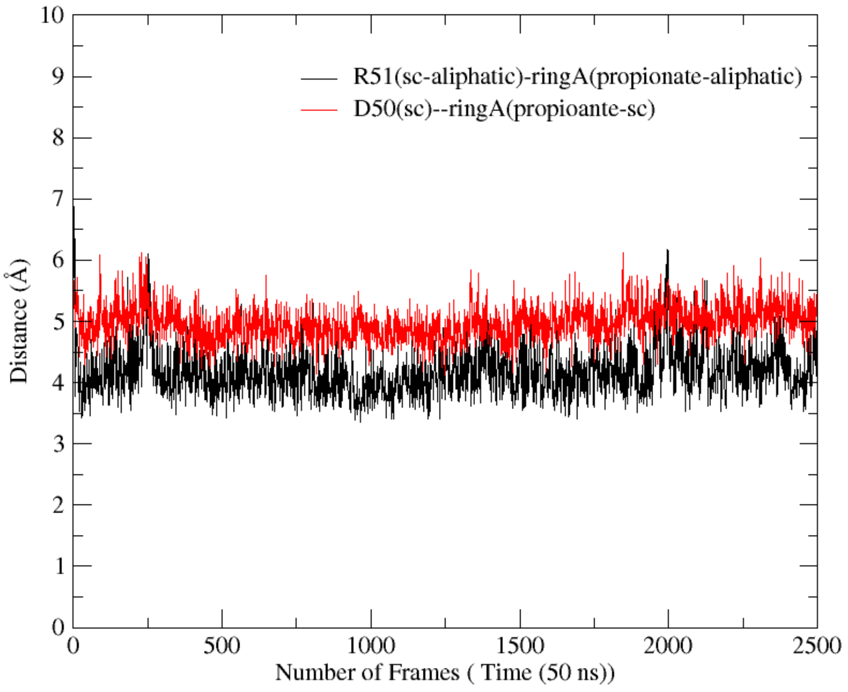

Figure S21 The interactions of the acetate side chain of ring A of UP2 with protein residue of WTFC

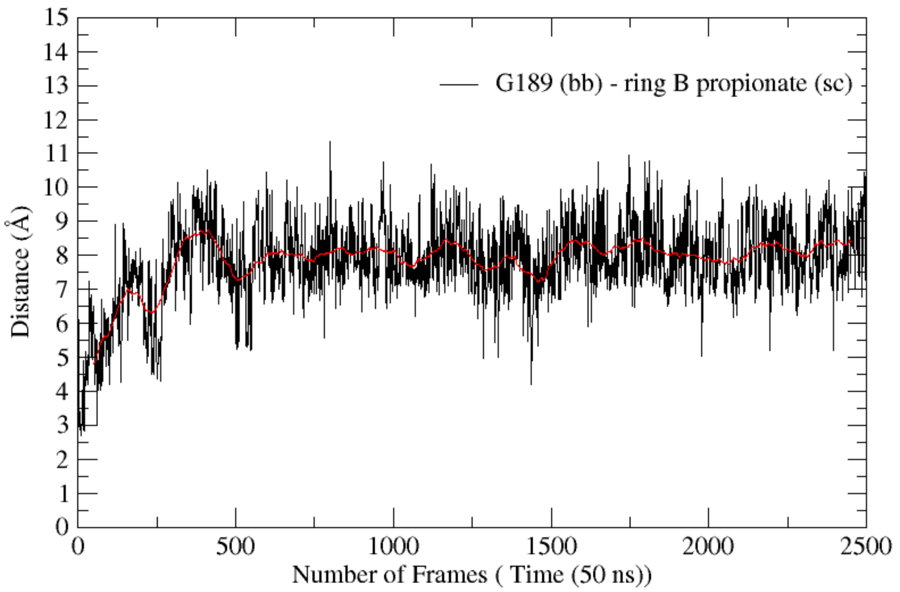

Figure S22 The interactions of the propionate side chain of ring B of the substrate UP2 with the G189 residue of WTFC

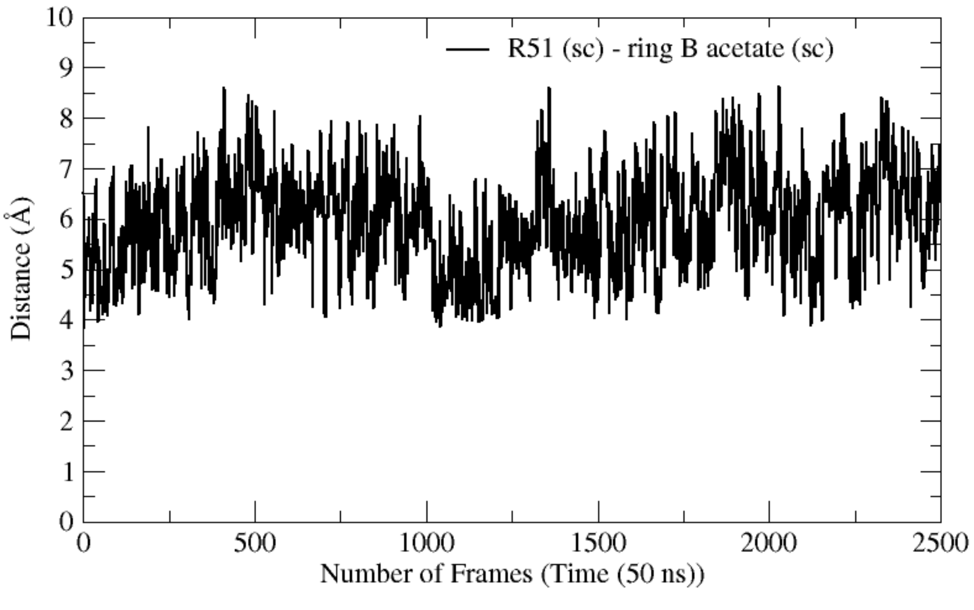

Figure S23 The side chain of the acetate of ring B of substrate interactions with the R51 residue of the WTFC

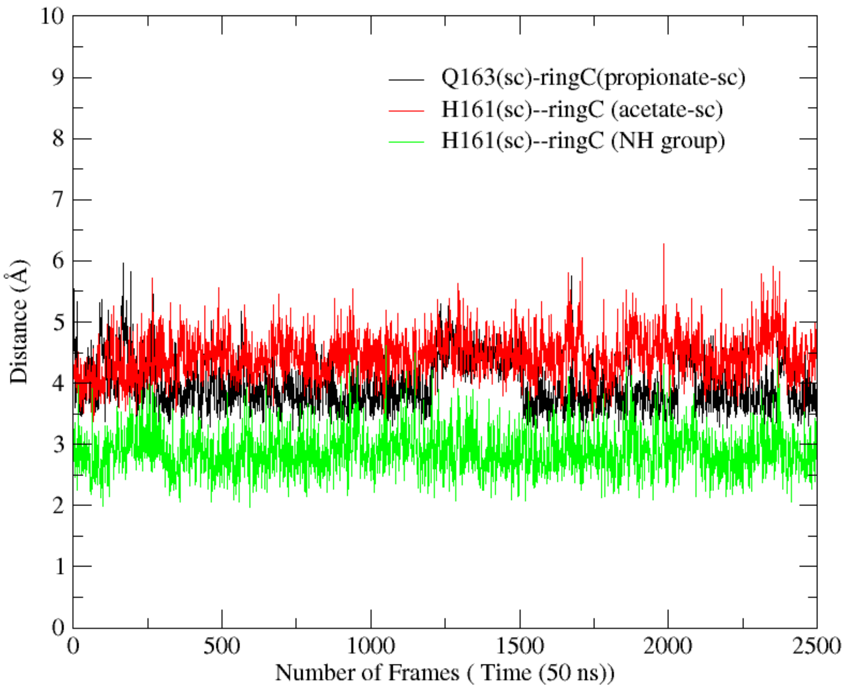

Figure S24 the interactions with the ring C of the substrate of substrate UP2 in WTFC

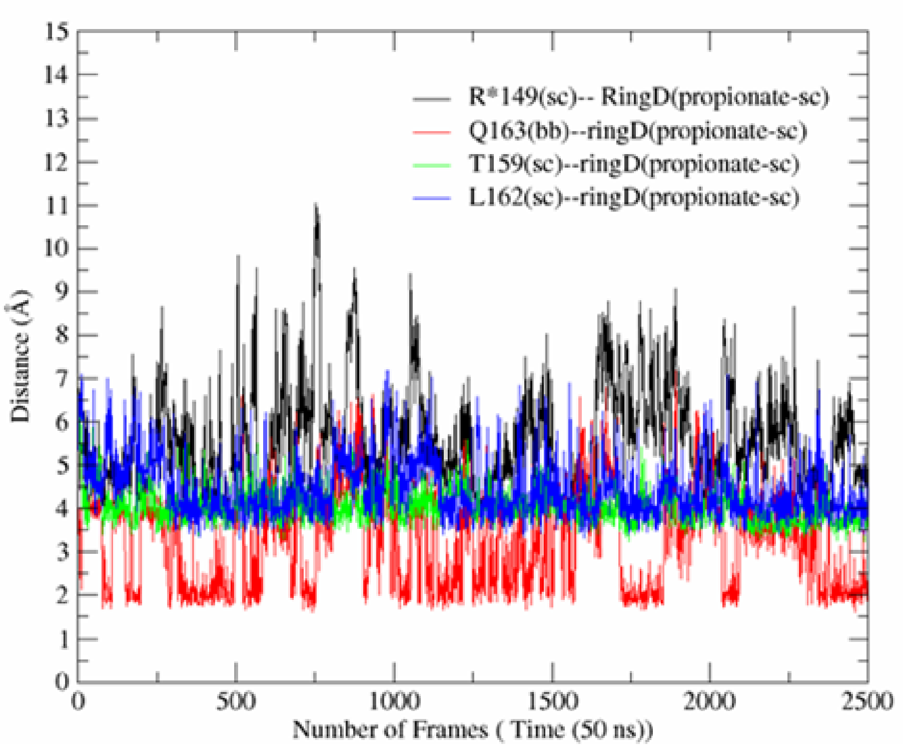

Figure S25 The interactions of the side chain of ring D proipionate of substrate UP2 in WTFC

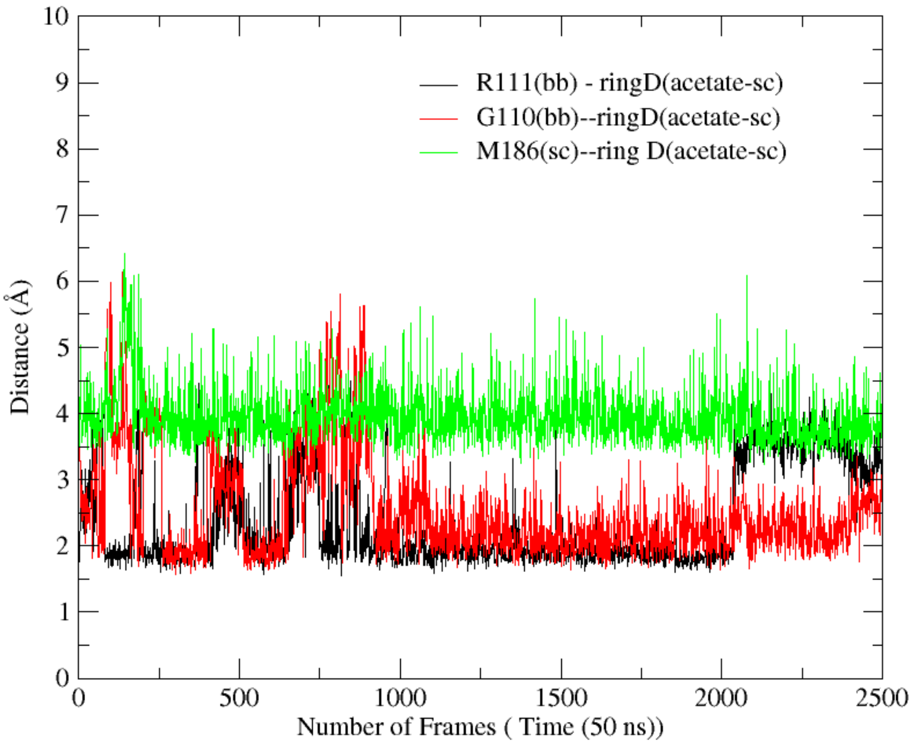

Figure S26 The interactions of ring D of substrate UP2 with residue of WTFC

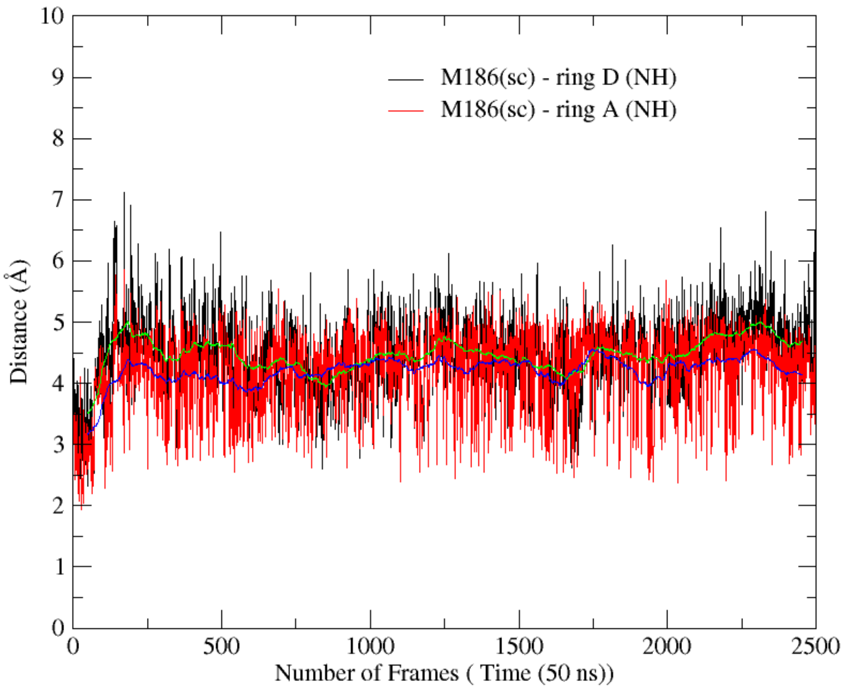

Figure S27 the hydrogen bonding of the M186 residue with the ring NH of substrate

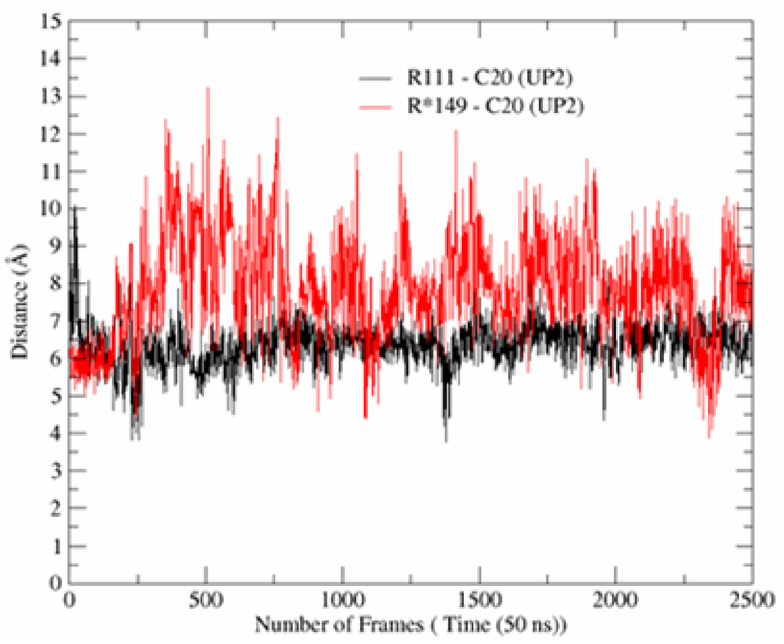

Figure S28 The interactions of the arginine R111 and R*149 of wild type NirE (WTFC) with the C20 potential proton abstraction site on substrate UP2.

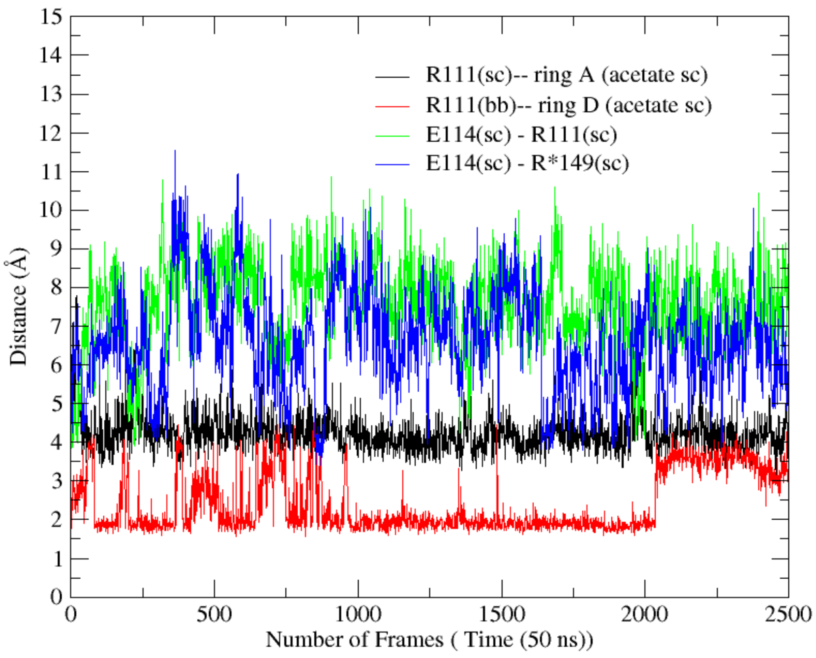

Figure S29 The interactions of R111 and E114 residues in the WT FC for 50 ns trajectory

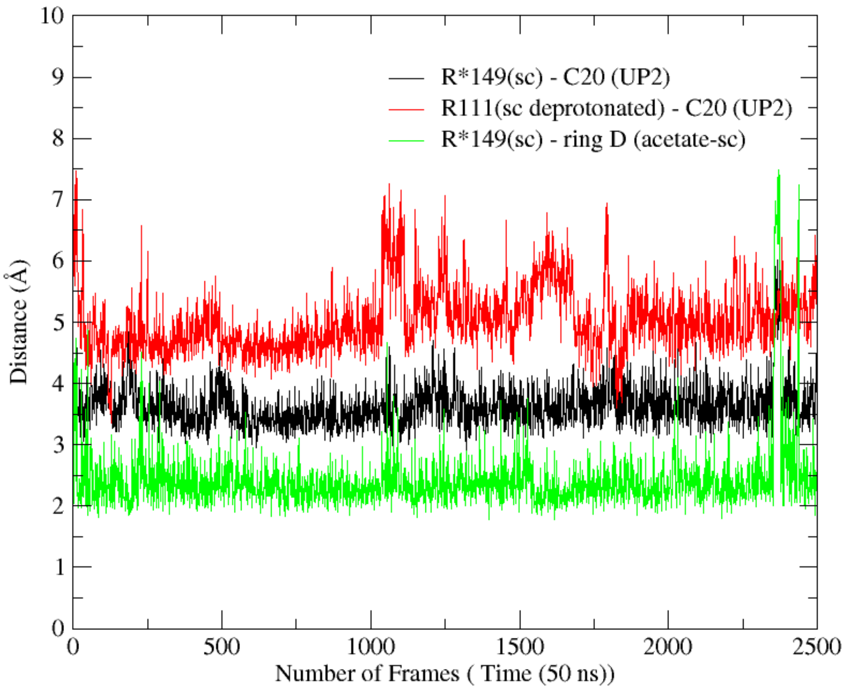

Figure S30 The MD simulation of R111 deprotonated setup and its important interactions with substrate UP2. Note here R*149 is in its normal state which is protonated (+1 charge).

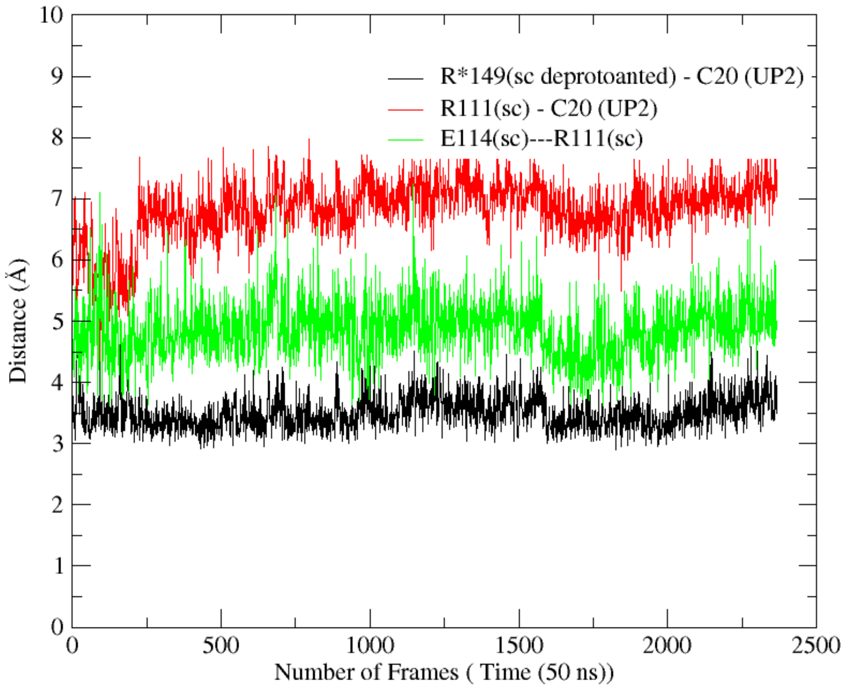

Figure S31 The MD simulation of R *149 deprotonated setup and its important interactions with substrate UP2. Note here the R111 is in its protonated normal state with chare of +1.

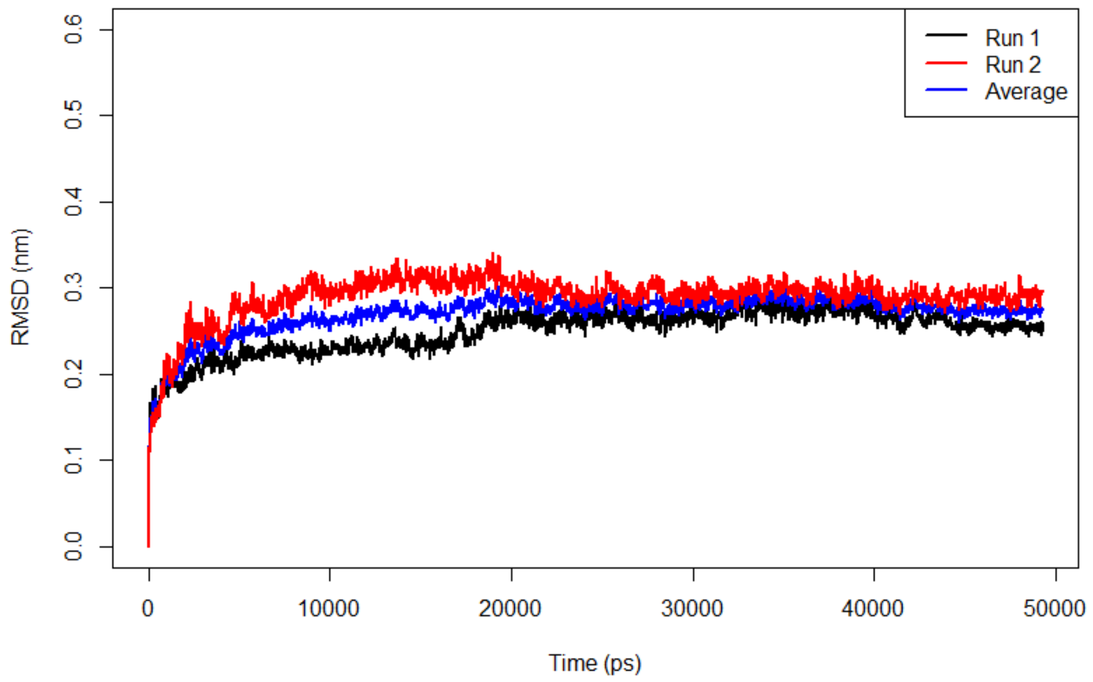

Figure S32 The deprotonated R*149 trajectory with average run for 50 ns. The average run is shown in blue colour.

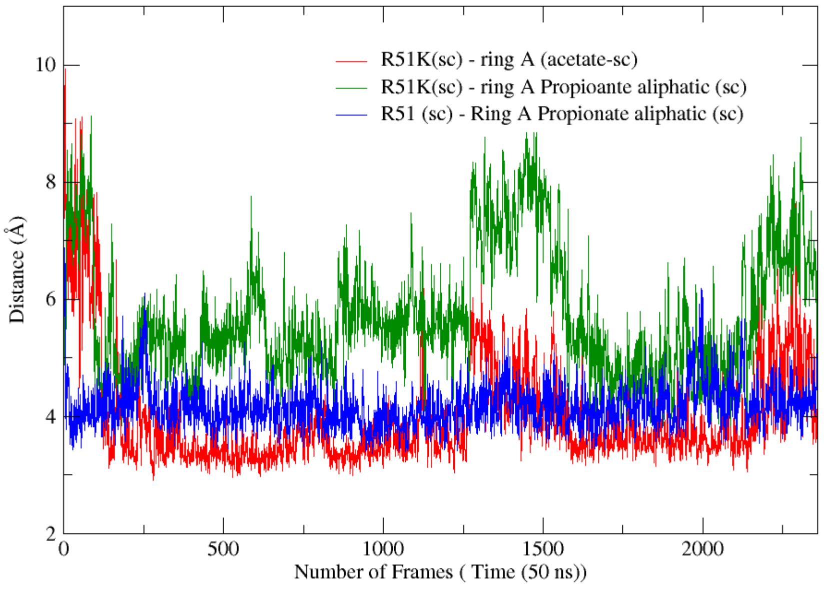

Figure S33 The interaction of mutant R51K with the substrate UP2

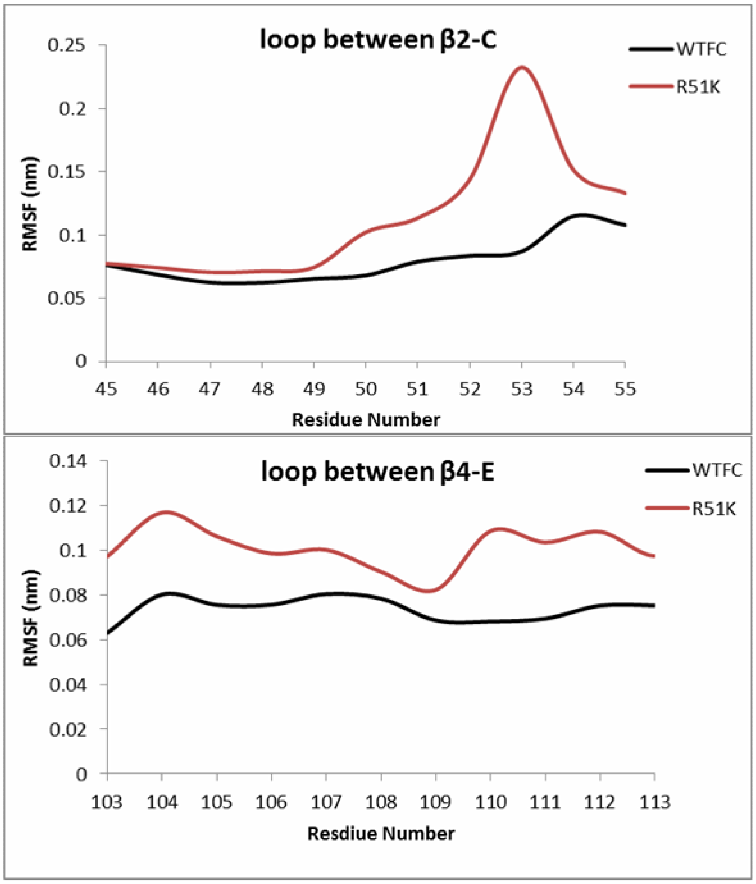

Figure S34 The RMSF value of the mutant R51K and the wild type NIRE

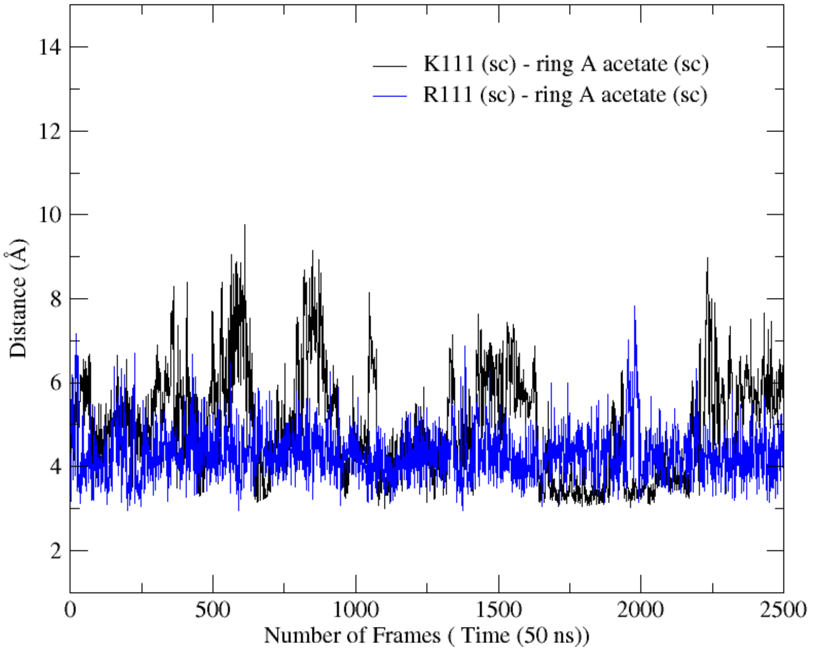

Figure S35 The interactions of the R111K mutant in comparison to wild type NIRE

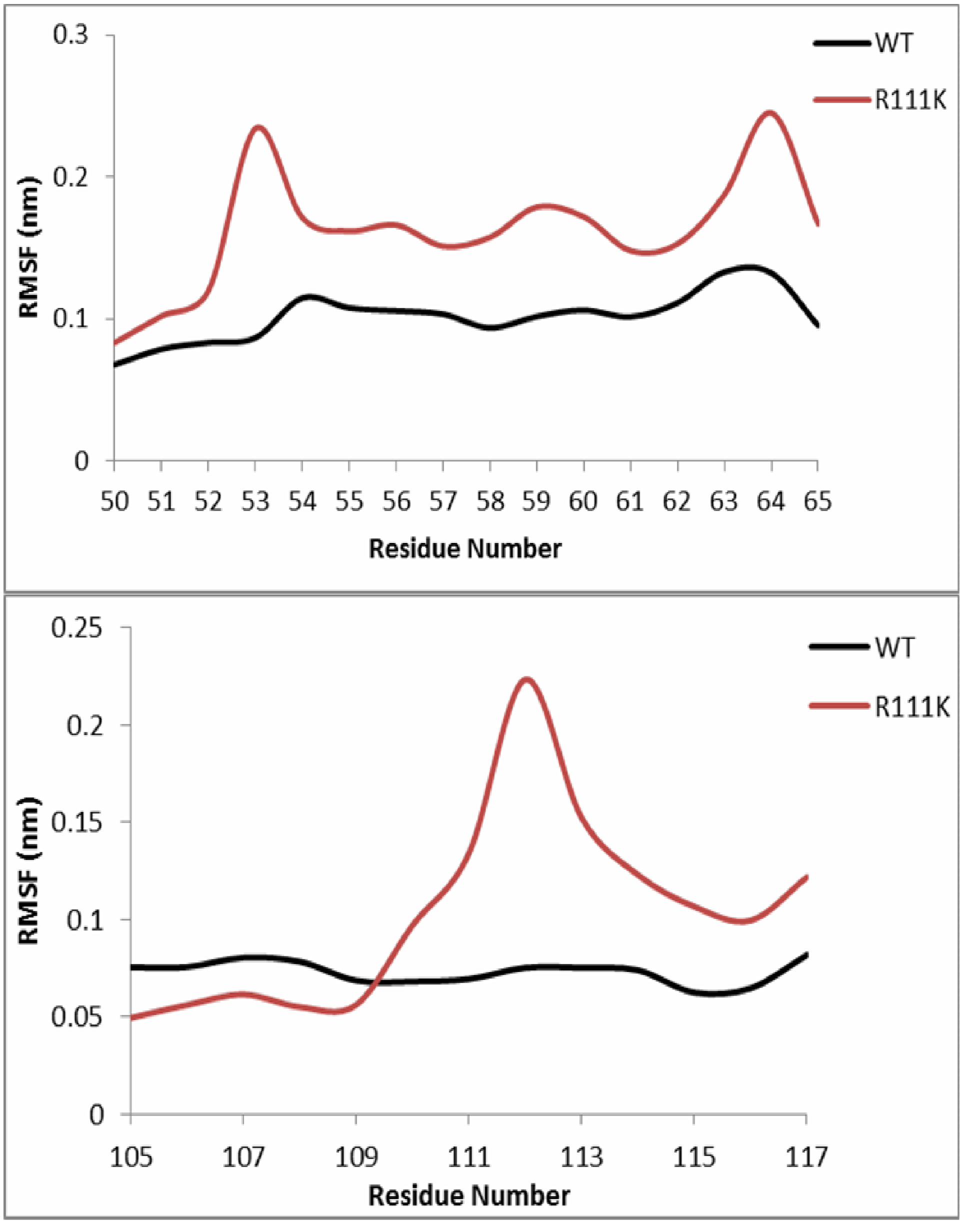

Figure S36 The residue show high RMSF in R111K mutant in contrast to WT FC.

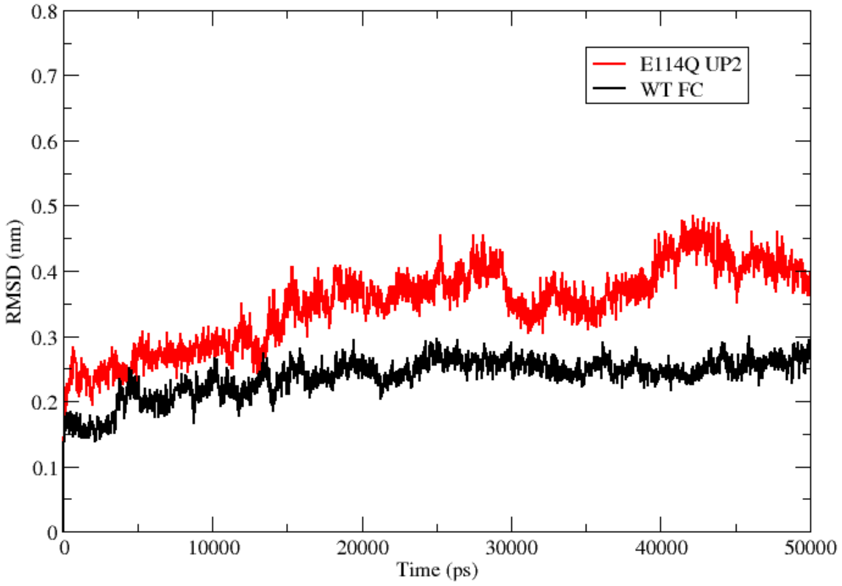

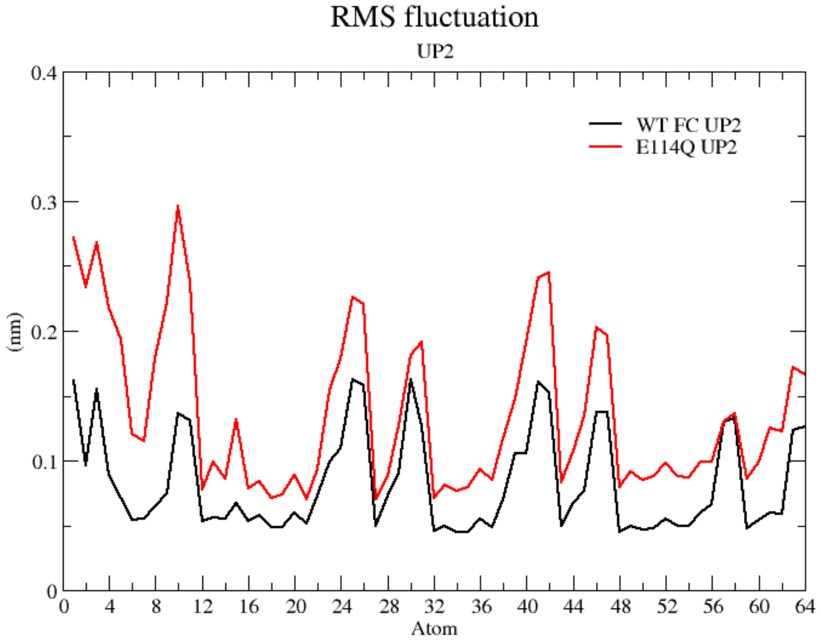

Figure S37 The RMSD and RMSF of substrate UP2 in mutant E114Q and WTFC

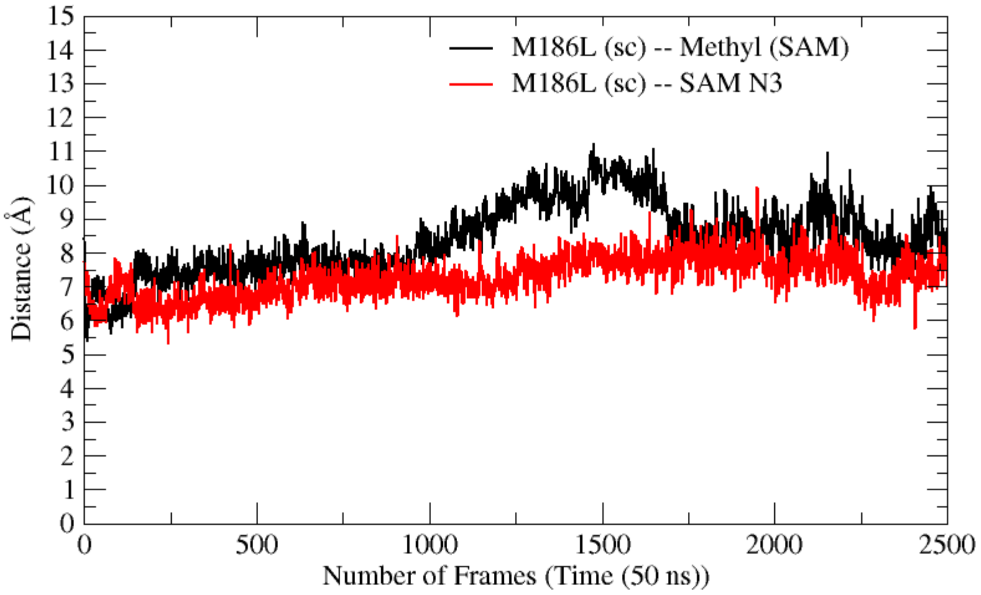

Figure S38 The interactions of the M186L mutant with cofactor SAM

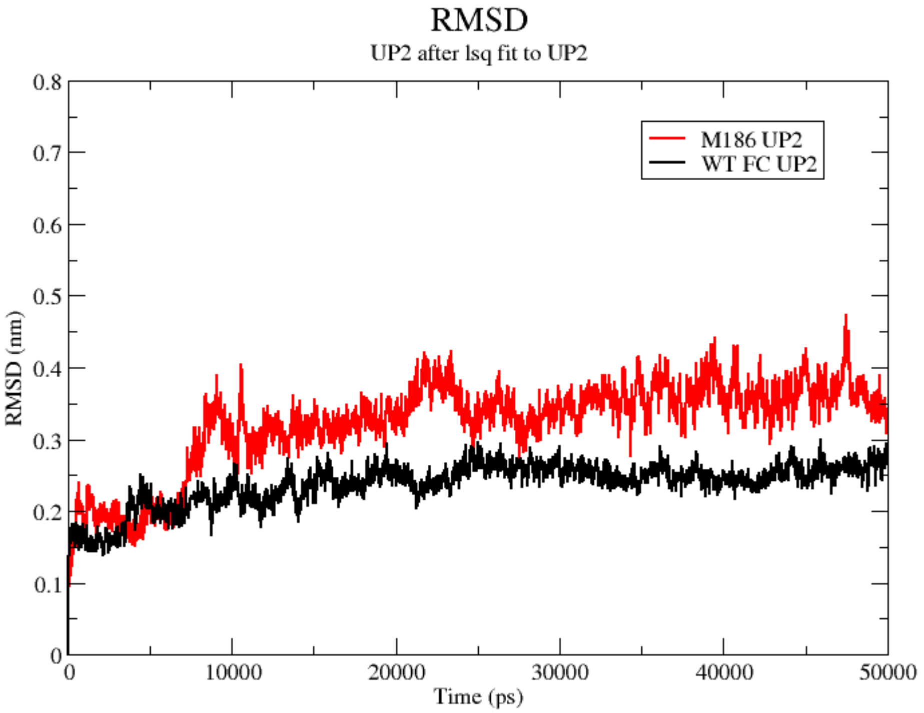

Figure S39 The RMSD profile of UP2 substrate using in M186L mutant

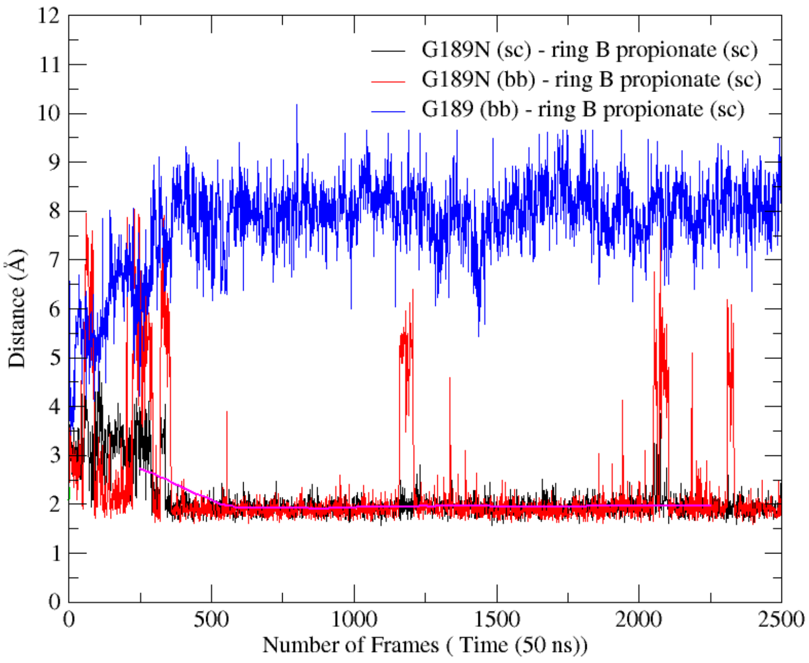

Figure S40 The interactions of the G189N mutant and comparison with WTFC G189 residue

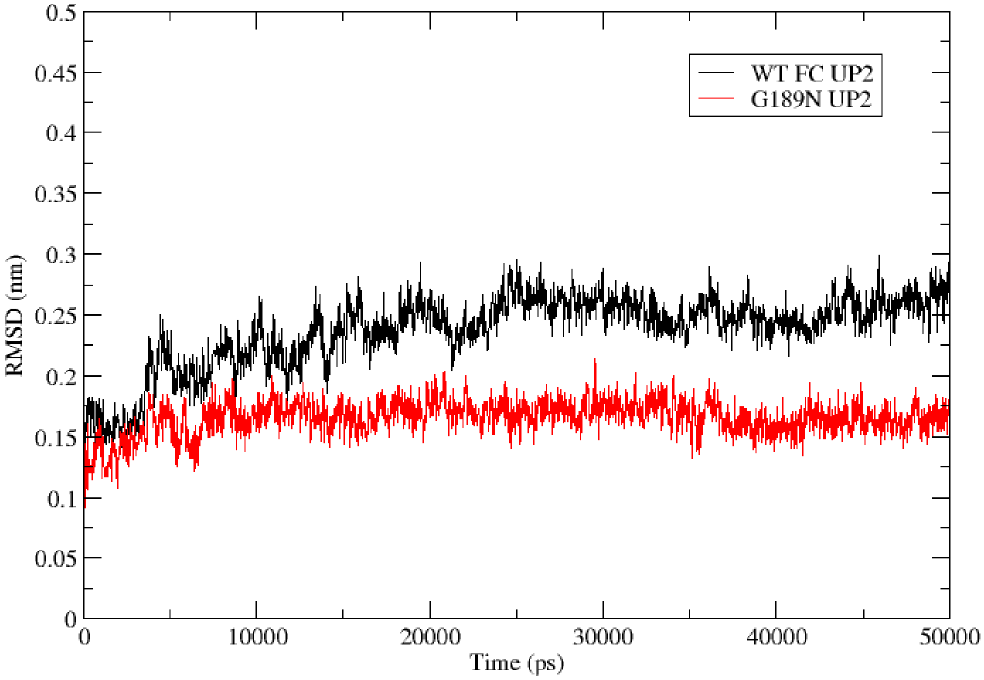

Figure S41 The RMSD of substrate UP2 in WTFC and mutant G189N for 50 ns trajectory

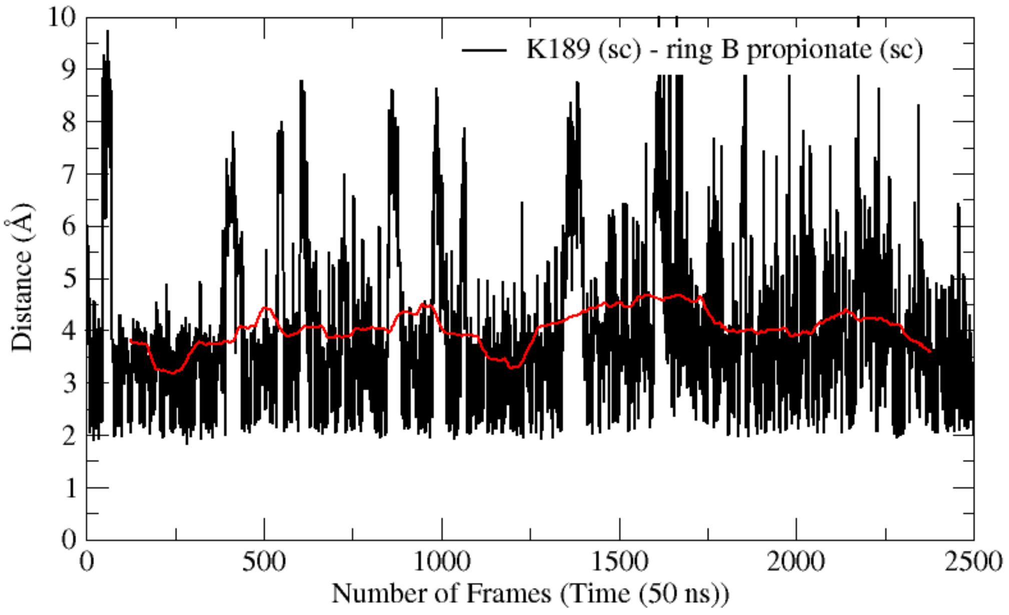

Figure S42 The electrostatic interaction of K189 in G189K mutant with the side chain of ring B of propionate of UP2

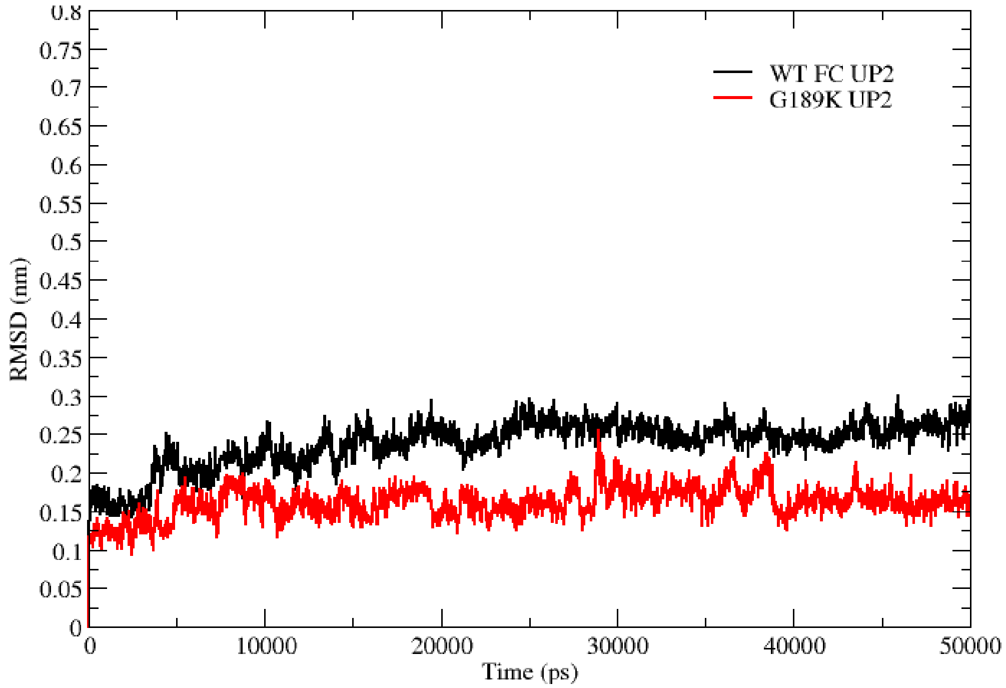

Figure S43 The RMSD of substrate UP2 for the WTFC and the G189K mutant

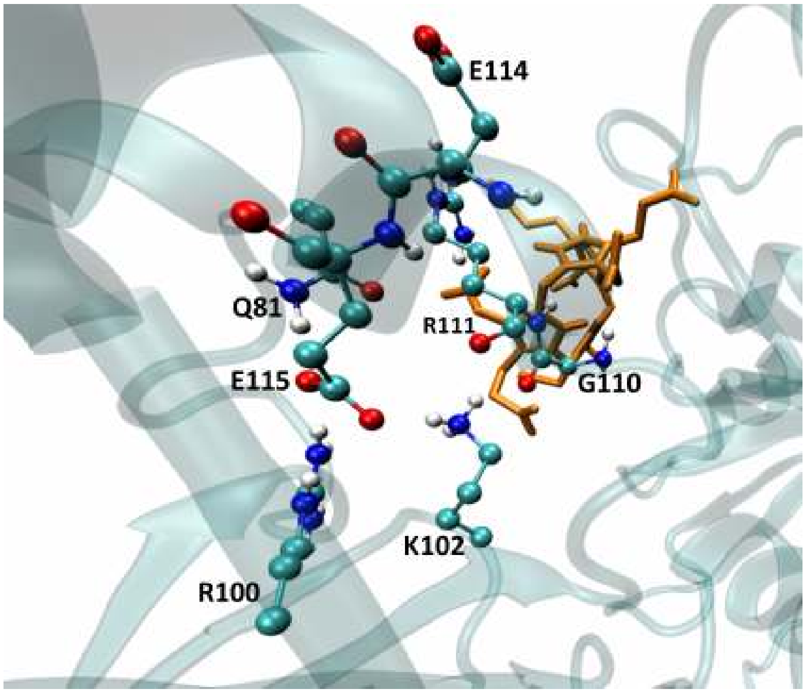

Figure S44 The interactions of K102 in the active site of the NirE wild type (WTFC) during MD simulation studies.

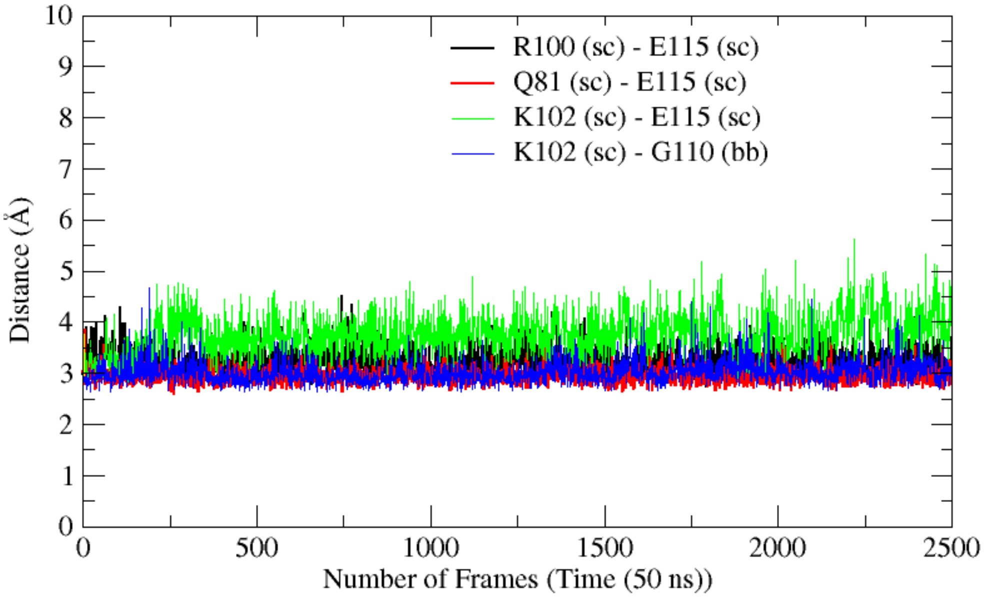

Figure S45 The interactions of K102 with the residues in vicinity of active site in WTFC

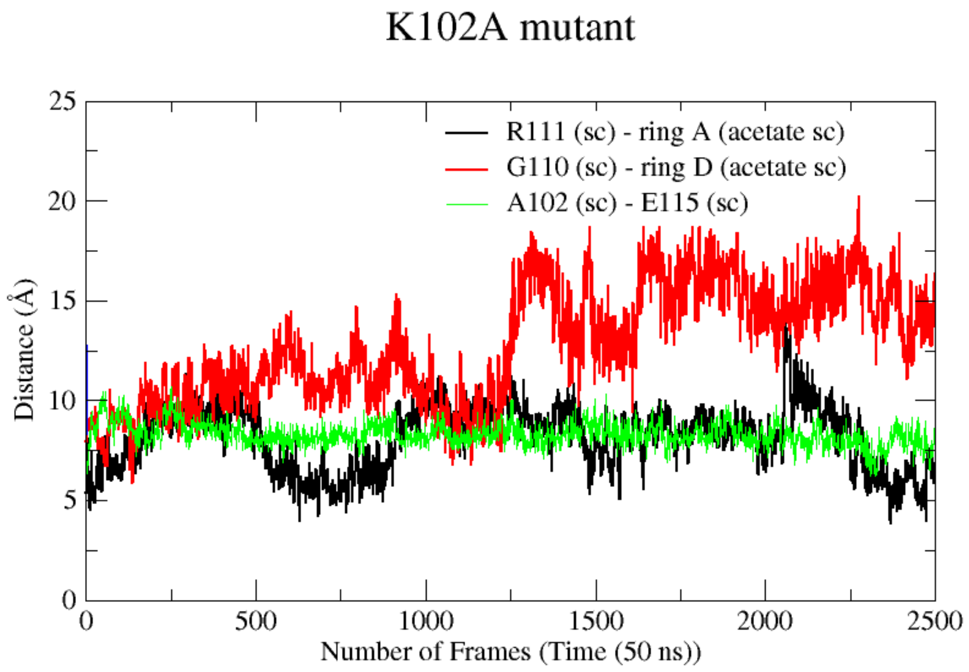

Figure S46 The interactions of K102A mutant.

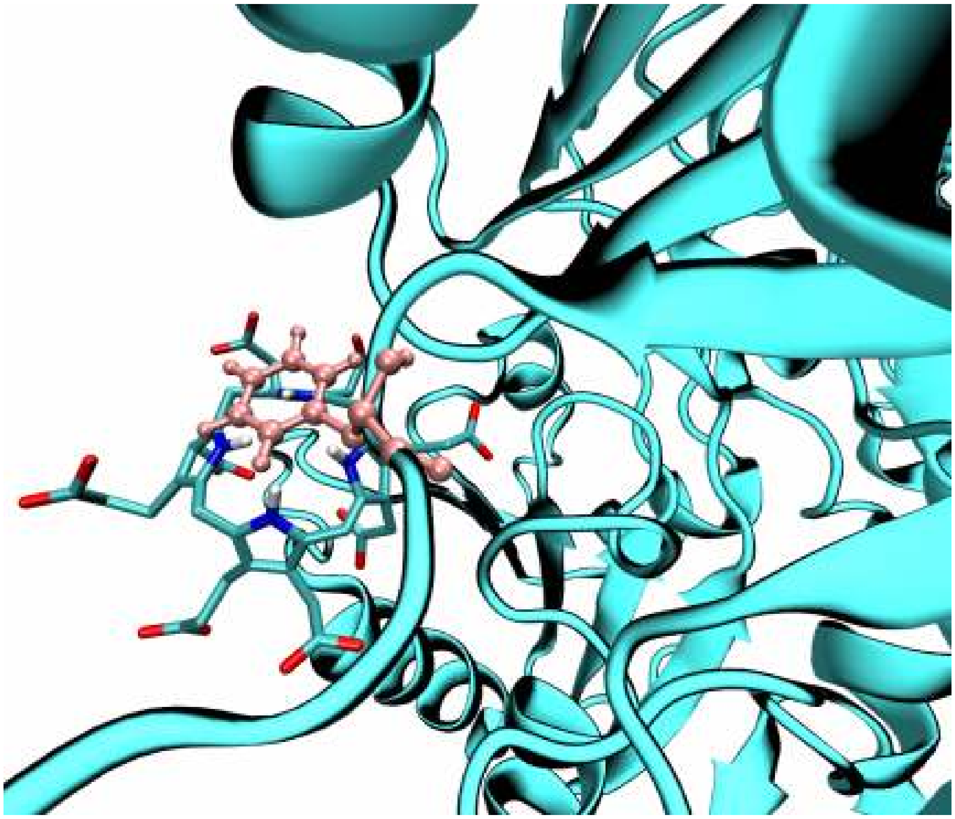

Figure S47 The head on overlap of the F161on UP22 in H161F mutant

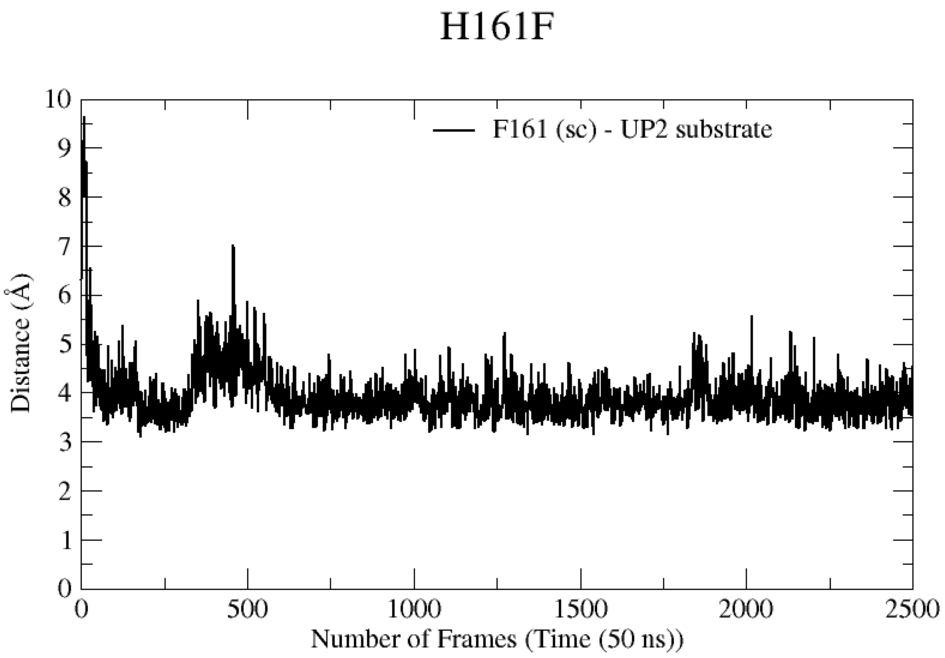

Figure S48 The pi pi stacking of the aromatic ring of the F161 in mutant with the UP2 ring system.

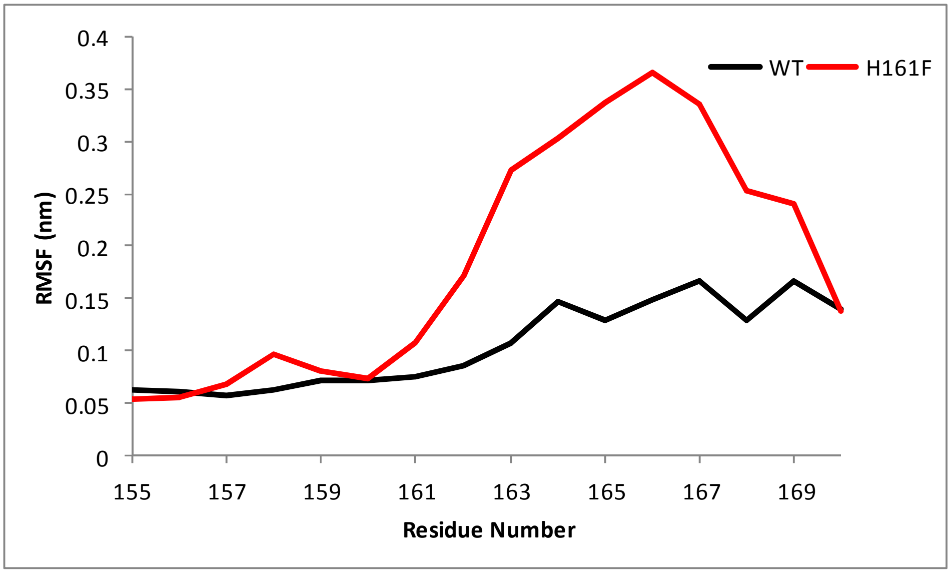

Figure S49 The RMSF of the local residue in vicinity of the mutant H161F

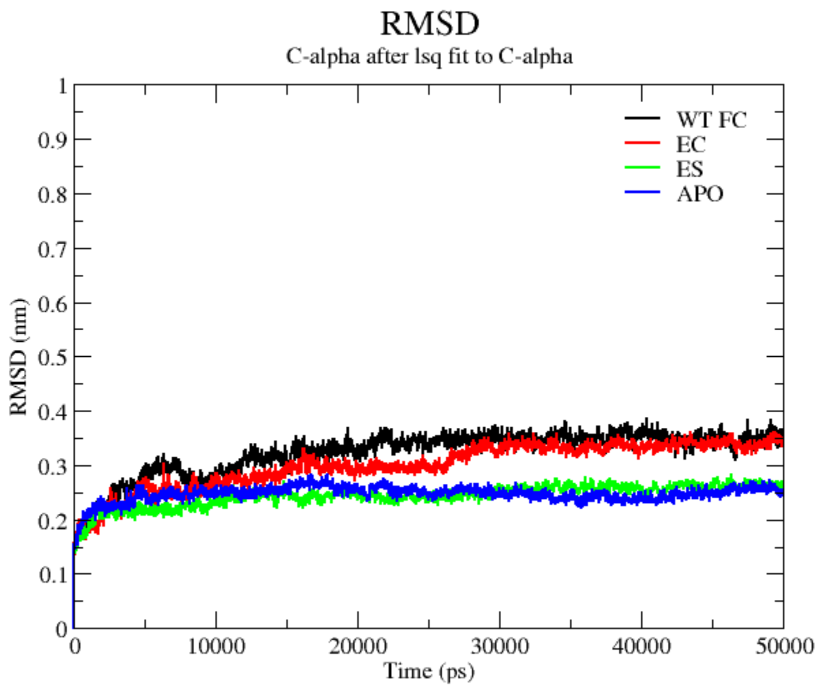

Figure S50 The RMSD of wild type NirE along with Apoenzyme, Enzyme substrate and Enzyme cofactor

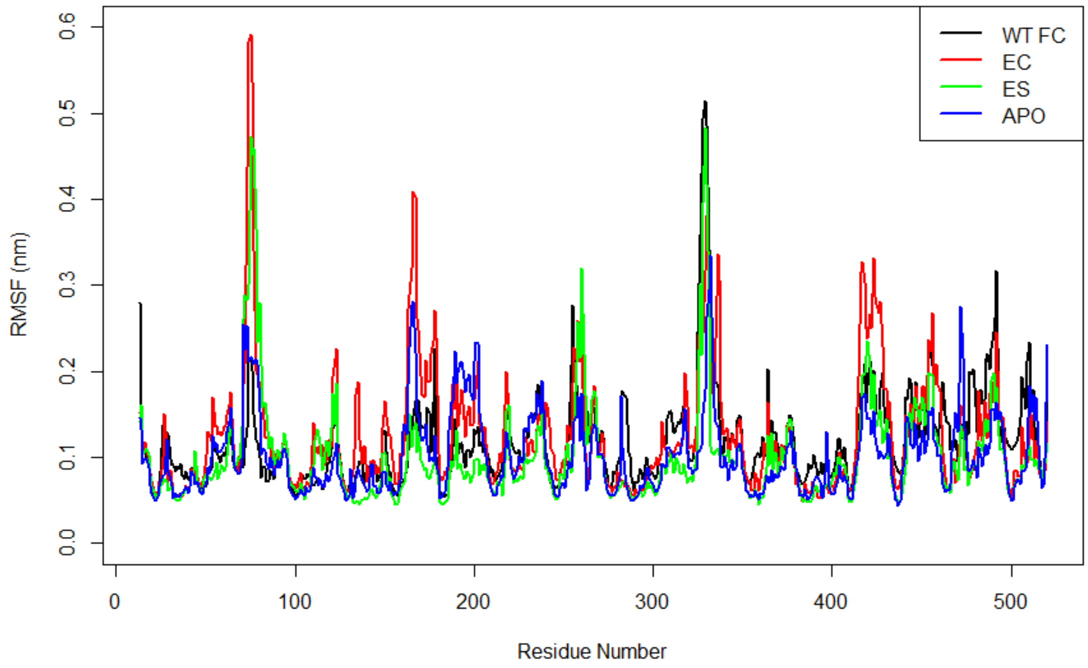

Figure S51 The RMSF plot of ES, EC, APO in comparison to WTFC using C α atoms for 50 ns simulation

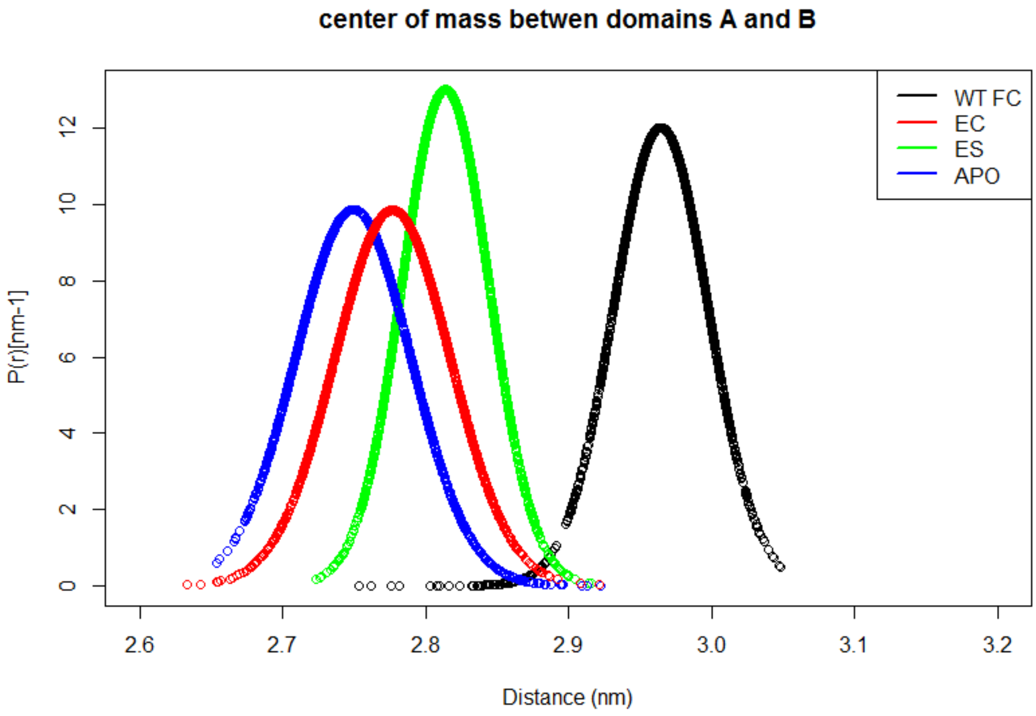

Figure S52 The normalized distrubtion of the centre of mass of domain A and B of monomer A of WTFC and the that of APO, ES and EC setups for 50 ns trajectory.

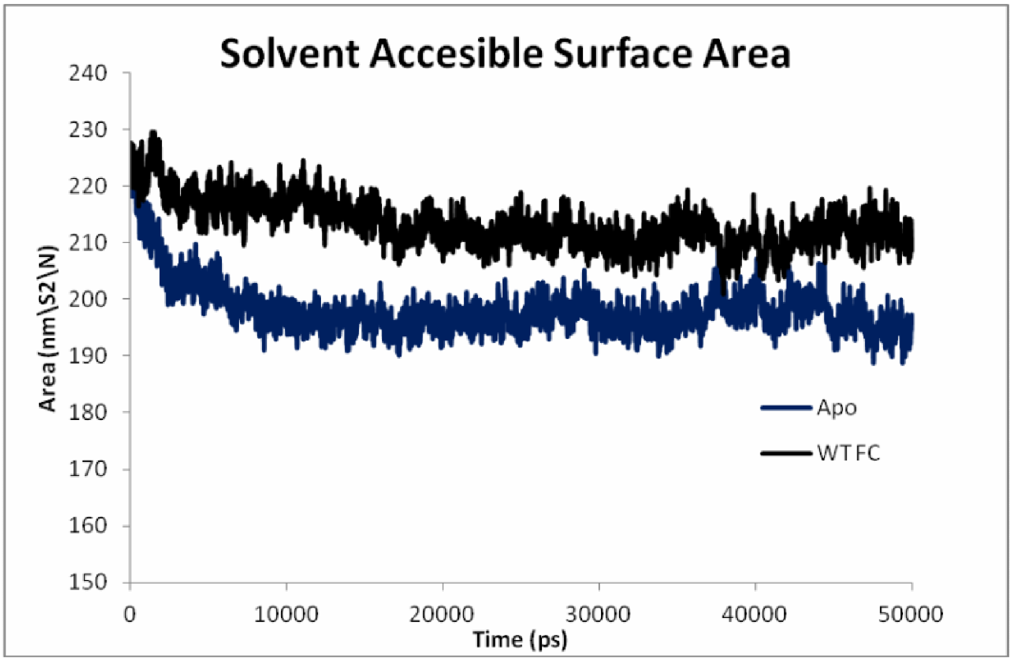

Figure S53 The solvent accessible surface area of the Apoenzyme and WTFC for 50 ns.

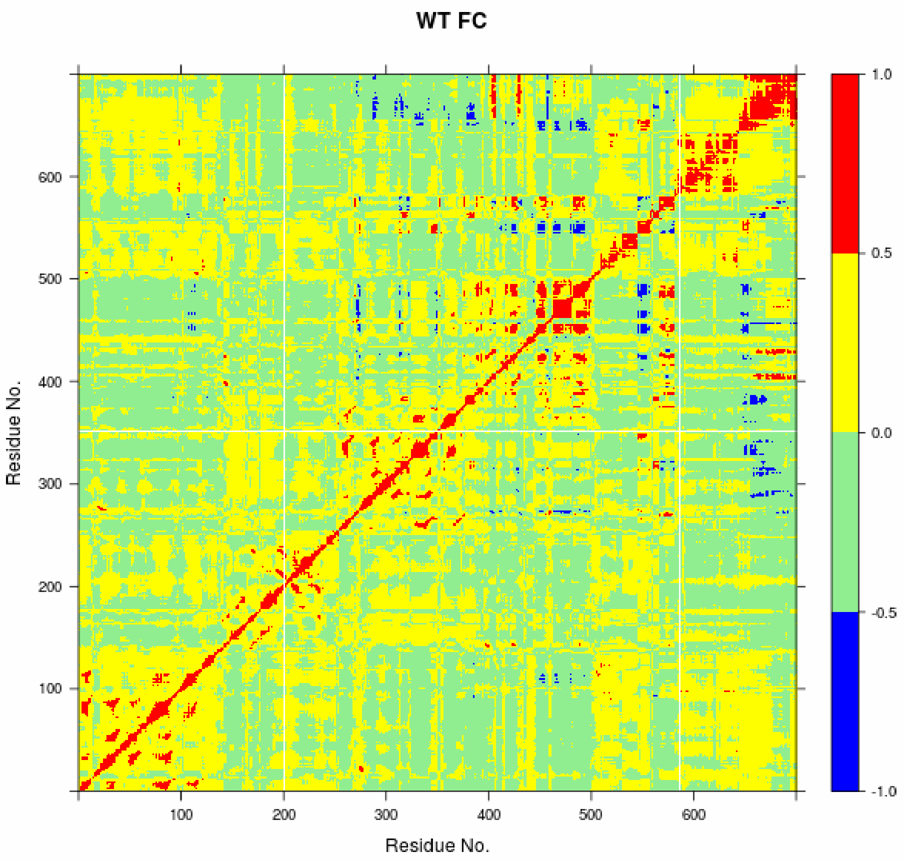

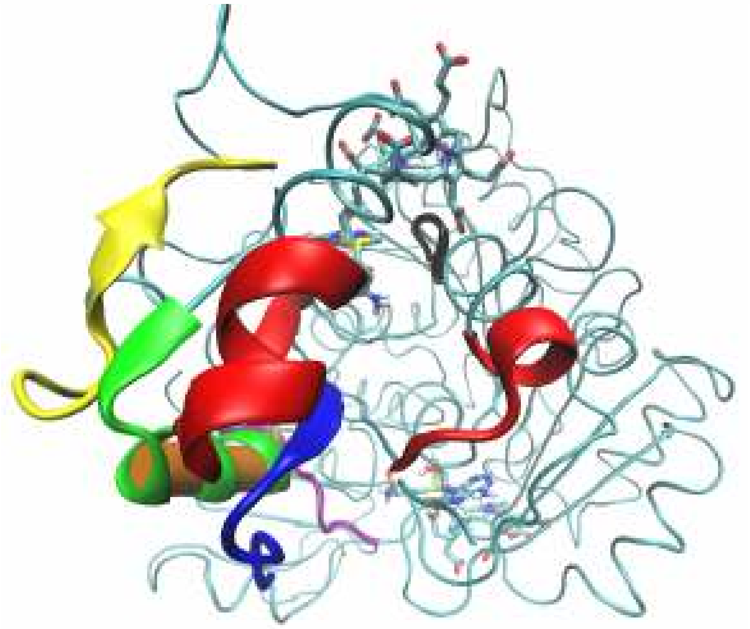

Figure S54 The Dynamic Cross Correlated motion analysis of the WT FC NirE for 50 ns. The correlated motion is color coded (1) blue is correlated to red color, (2) green to yellow color, (3) orange to purple,4 black color represent correlated motion towards the substrate UP2 atoms especially the side chains. The plots were made using Bio3D package in R

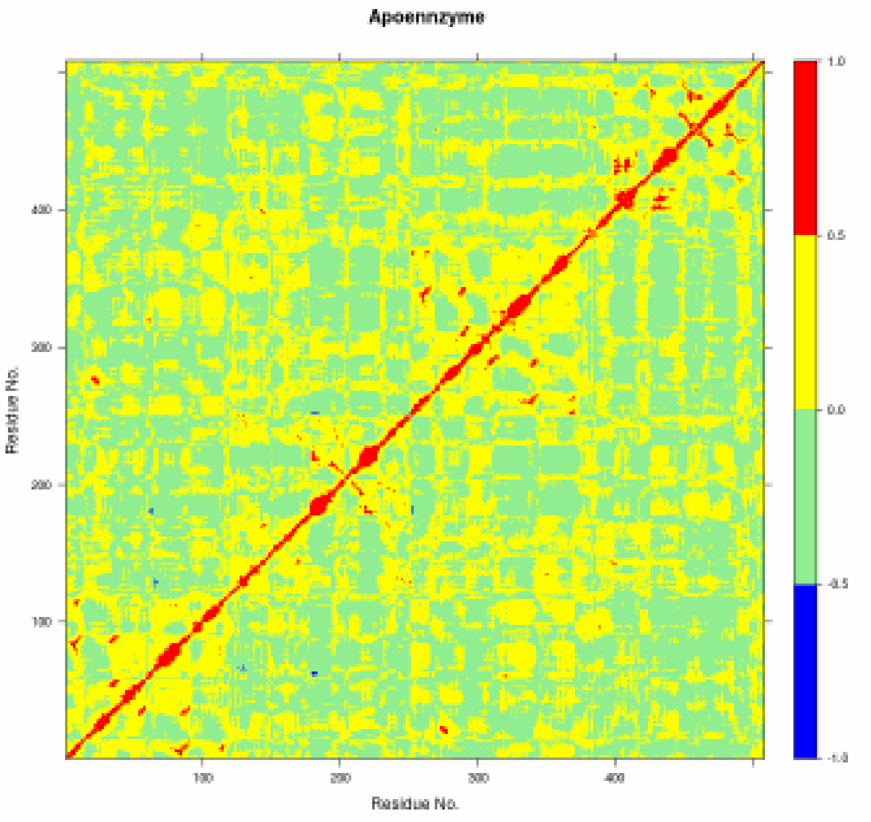

Figure S55 The Dynamic Cross Correlated motion analysis Apo enzyme for 50 ns

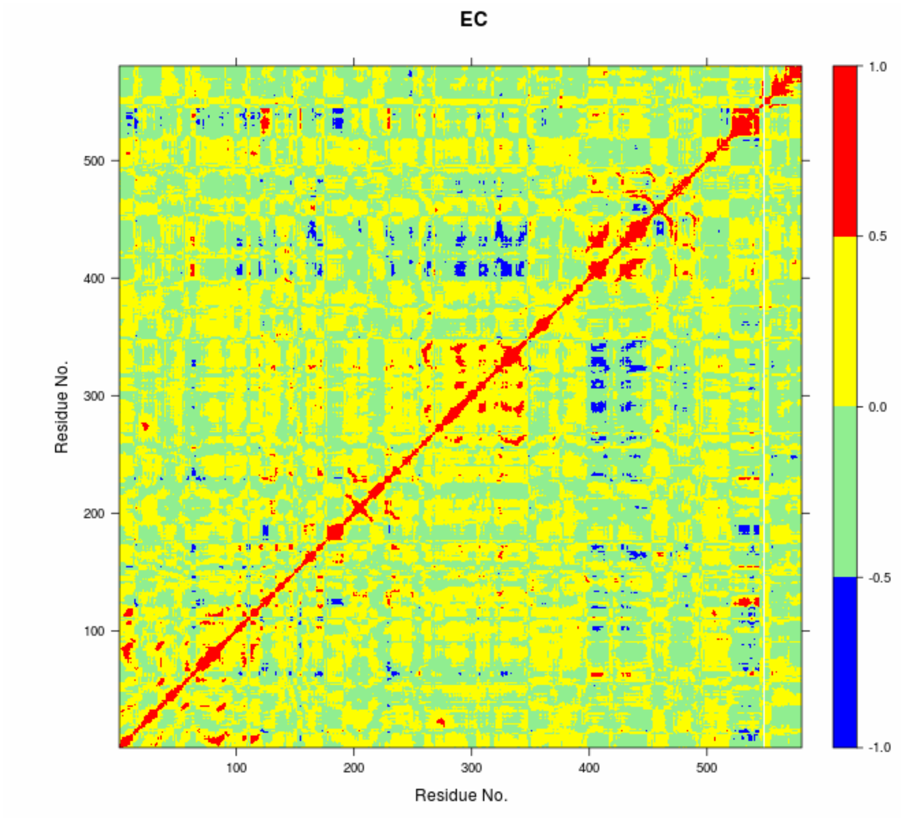

Figure S56 The Dynamic Cross Correlated motion analysis of EC complex

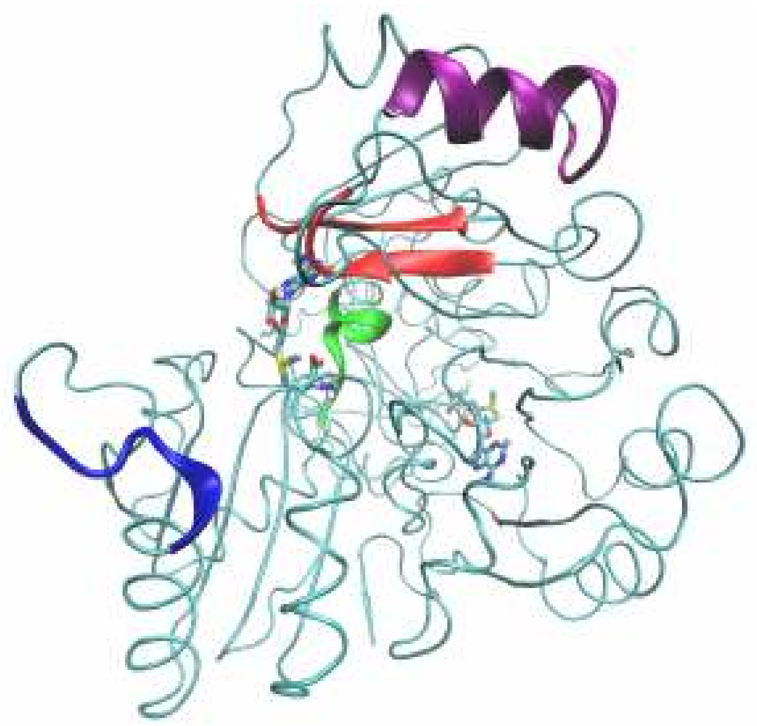

Figure S57 The anti-correlated motion is colour coded (1) blue is correlated to red colour, (2) red to red colour, (3) green to purple colour

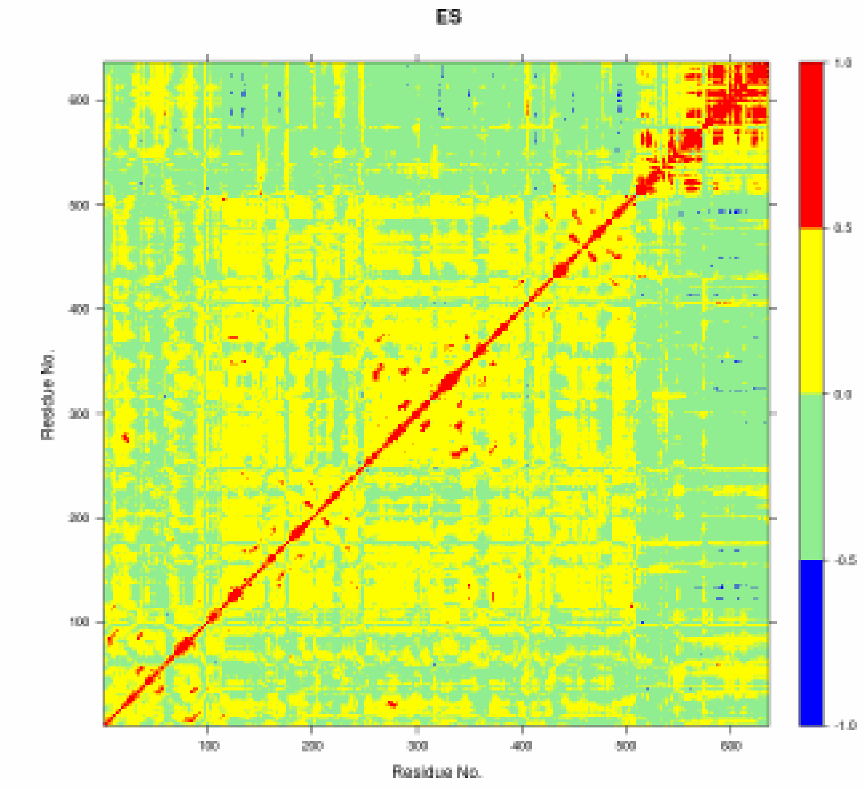

Figure S58 The Dynamic Cross Correlated motion analysis of ES complex
